# Automated Remote Pulse Oximetry System (ARPOS)

**DOI:** 10.3390/s22134974

**Published:** 2022-06-30

**Authors:** Pireh Pirzada, David Morrison, Gayle Doherty, Devesh Dhasmana, David Harris-Birtill

**Affiliations:** 1 School of Computer Science, University of St Andrews, St Andrews KY16 9AJ, UK; dm236@st-andrews.ac.uk (D.M.); dcchb@st-andrews.ac.uk (D.H.-B.); 2 School of Psychology and Neuroscience, University of St Andrews, St Andrews KY16 9AJ, UK; ghm@st-andrews.ac.uk; 3 School of Medicine, University of St Andrews, St Andrews KY16 9AJ, UK; djd5@st-andrews.ac.uk; 4 Department of Respiratory Medicine, Victoria Hospital, NHS Fife, Hayfield Road, Kirkcaldy KY2 5AH, UK

**Keywords:** remote health monitoring, heart rate measurement, blood oxygenation level measurement, rPPG system

## Abstract

Current methods of measuring heart rate (HR) and oxygen levels ($SPO_{2}$) require physical contact, are individualised, and for accurate oxygen levels may also require a blood test. No-touch or non-invasive technologies are not currently commercially available for use in healthcare settings. To date, there has been no assessment of a system that measures HR and $SPO_{2}$ using commercial off-the-shelf camera technology that utilises R, G, B, and IR data. Moreover, no formal remote photoplethysmography studies have been performed in real-life scenarios with participants at home with different demographic characteristics. This novel study addresses all these objectives by developing, optimising, and evaluating a system that measures the HR and $SPO_{2}$ of 40 participants. HR and $SPO_{2}$ are determined by measuring the frequencies from different wavelength band regions using FFT and radiometric measurements after pre-processing face regions of interest (forehead, lips, and cheeks) from colour, IR, and depth data. Detrending, interpolating, hamming, and normalising the signal with FastICA produced the lowest RMSE of 7.8 for HR with the r-correlation value of 0.85 and RMSE 2.3 for $SPO_{2}$. This novel system could be used in several critical care settings, including in care homes and in hospitals and prompt clinical intervention as required.

## 1. Introduction

Heart rate (HR), oxygenation levels ($SPO_{2}$), body temperature (BT), and blood pressure (BP) are critical physiological signals that reflect human health [1]. For example, in cardiovascular disease, a decrease in HR may reflect a rise in intracranial pressure [2], whilst an increase in HR may reflect hypovolaemic shock [3]. Blood oxygen levels represent a key parameter of respiratory health and low levels may signal respiratory failure [4]. For healthy adults, the heart beats at about 60 to 100 beats per minutes (BPM) and pumps blood through the body. Tachycardia refers to an abnormal HR, which is above 100 BPM, and bradycardia to an abnormally slow HR below 60 BPM [5].

Traditionally, finger probes and blood pressure cuffs using the principles of photoplethysmography (PPG) are most commonly used to measure vital signs, including blood oxygen saturation, heart rate, blood pressure, and cardiac output [6,7,8]. Another widely utilised form of monitoring is the electrocardiogram (ECG), which involves wearing gel patches or straps around the chest. Different types of wearable devices are also available to measure these vital signs, which can be worn on different parts of the body such as the wrist, ankle, earlobe, etc. [9]. Blood oxygen levels are currently assessed either by simple methods such as a finger probe placed around the tip of the finger or by more invasive procedures such as blood tests, typically, a needle into an artery in the wrist [10]. The former technique although simple is open to significant error and fluctuation, whilst the latter is painful and carries significant risks. A $SPO_{2}$ value above 94% is considered to be normal for most individuals, whilst some people with stable chronic lung disease may have an acceptable value of 88-94%. Values below approximately 94% usually correspond to respiratory failure and would indicate a significant respiratory illness [11]. The most common way to measure both HR and $SPO_{2}$ simultaneously is with a pulse oximeter (clinical or commercial). It is a simple, non-invasive, and widely accepted device used in clinics and hospitals. A pulse oximeter uses PPG principles to obtain these vitals. Hertzman introduced PPG in 1937 [12], PPG measures blood flow by calculating changes in the dispersion of `photo’ (light) [8]. It uses a photo-diode and two different light sources [13]. A clip is placed on the patient’s finger and the light source emits light to the finger. The photodetector measures the reflectance of light from this finger to measure the vital signs [8].

Although effective, pulse oximetry poses several problems as a monitoring tool. The pulse oximeter clip has to be physically mounted on the finger of a patient, which can be difficult to manage for adults with any form of incapacity, common in the elderly, young children, and in those with severe illness of any cause [14]. Moreover, this presents a potential hazard with a high risk of choking among infants when using such devices [15]. It may be burdensome and uncomfortable if the patients have to wear it for a prolonged time period, may restrict movements, carries increased risk of cross-infection, and becomes a constant reminder of their ill-health and of being monitored continuously [16]. In addition, the value in carrying out these observations is in prompt recognition of any abnormal signs. It is important to rapidly triage patients who are at highest risk of health decline in clinical settings. In this context, there would be significant advantages in having remote sensing technology that simultaneously and in real-time tracks multiple patients’ vital signs. This would enable the immediate recognition of emerging abnormal vital signs, and so enable the subsequent provision of rapid intervention. This is in contrast to current manual methods which are both slow when taken per patient, and staff-dependent requiring manual input into the relevant recording system before any responder action is possible.

These problems can be avoided by using remote photoplethysmography(rPPG) monitoring systems. rPPG is the measurement of the flow of blood by optical means, typically involving measurement of changes in the transmission or scattering of light created by blood flow in a part of the body [15]. These changes are also reflected on the face via subtle changes on the skin where the pulse flashes lighter and darker over time. This phenomenon is not visible to the naked eye; however, it can be detected by measuring the reflectance of light over a period of time from skin pixels [17]. The rPPG system measures the heart rate by analysing the skin pixel intensity of the heart beats over time; the skin flashes darker and lighter as more and less blood flows through the region. It measures the oxygenation level by using the optical absorption differences across the visible and near-infrared wavelength regions between oxy-haemoglobin (Hb) and deoxy-haemoglobin (deoxy-Hb) [18].

## 2. Previous Work

Monitoring health remotely, specifically vitals, has been an area of interest for many researchers. Most of the research focuses only on HR, whereas only a limited number of studies focus on $SPO_{2}$. The system performance metrics (RMSE, r-correlation, and standard deviation error values) of previous work are listed in Table 1 for HR and Table 2 for $SPO_{2}$.

Early research in this area detected HR using a laptop camera to acquire face colour images consisting of red (R), green (G), and blue (B) channels. Independent component analysis (ICA), fast Fourier transform (FFT), and a frequency filter were applied on the channel data (R, G, B) to obtain the pulse signal. The second component (green channel) was found to typically produce the strongest pulse signal in this study [19]. This research was further refined by adding signal pre-processing such as detrending and smoothing the signal data. The system was updated to select a component with the highest peak after applying FFT and filter, which was further smoothed by moving average filter [22]; however, the study was conducted within a lab setting in a controlled environment with only a limited number of participants (*n* = 12).

Studies have also used machine learning techniques to measure remote HR where the researchers [23] expanded on the work performed by Poh et al. [19]; however, the accuracy drops for participants in motion and under different light sources. To cater to participant movement, the researcher further improved the system by applying power spectrum analysis, K-nearest neighbors (KNN), and linear regression to each component extracted after applying ICA to obtain features and classify them [23]; however, generalising the model among the participants can bear different results or not perform well when new participants are introduced to the system on which it has not been trained yet and may fail in a real-life scenario. As with the previous study, this was conducted in a controlled environment with specific light conditions.

To cater to challenges associated with different illumination and participant movement, Li et al. [24] suggested a technique based on normalised least mean square (NLMS) adaptive filtering and face tracking using the Viola–Jones face detector [30] to overcome these challenges; however, the database used contains data obtained in a controlled environment with only slight movement among the participants. ROI tracking also fails when participants move at an extensive angle and participants’ facial expressions creates noise, which then shows high variations in the obtained signal. To increase signal-to-noise ratio (SNR), one of the studies suggested using a monochromatic camera with a green range filter. They also used weighted average on the various face regions of interest (ROI). A deformable face fitting algorithm and Kanade-Lucas Tomasi (KLT) tracking [31] algorithm were used to track and extract face features [25]; however, the RMSE obtained from this research is very high as mentioned in Table 1, which shows that it is not a feasible method to apply in a real-life system. A recent study proposed a method that uses Eulerian video magnification (EVM), quality assessment (QA) of signal, and adaptive chirp model decomposition (ACMD) to obtain HR. They validated the system in different illuminations and with some head motion [26]; however, once again, the setup was within a controlled setting where participants were at a distance of 0.6 m from the camera. The participant diversity information was not revealed. In addition to that, only the forehead is selected as ROI, which would fail if the participant is not facing the camera.

Researchers most commonly used principal component analysis (PCA) [32,33], ICA [34,35,36,37,38,39], FastICA [24,35,36,38,39,40,41,42,43,44,45,46], RobustICA [47], and joint approximation diagonalisation of eigen-matrices (JADE) [48] algorithms for extracting source signals from R, G, B channels to obtain HR. One paper used a Laplacian eigenmap (LE) [49] to extract signal sources from the face data obtained from participants. LE is also a dimensionality reduction technique but is non-linear to find the internal structure of the data. The research revealed better results in comparison to other dimensionality reduction techniques; however, this has not been tested on a larger dataset obtained from a real home environment.

There are far fewer studies on methods to obtain $SPO_{2}$. A new method to cancel aliased frequency components induced by fluorescent light flickering has previously been proposed based on autoregressive (AR) modelling and pole cancellation, which improved the effectiveness of the method under fluorescent illumination [50]. The research was conducted on patients undergoing kidney dialysis (in resting state with minimal movement) [50]; however, movement and illumination change increased noise, in turn impacting the system accuracy. The research also does not provide RMSE, r-correlation, or standard deviation error for $SPO_{2}$. Using a webcam, another study measured $SPO_{2}$ and HR by using an algorithm for noise removal based on dual-tree complex wavelet transform (DTCWT) to fix motion artefacts and artificial illumination [6]. Researchers also used R, G, and B channels to obtain $SPO_{2}$ by assessing the pulse signal at two wavelengths of 660 nm and 940 nm [51]. This was obtained by comparing red and blue wavelength bands; however, these studies [6,28,51], only used a 0.5 m range, which is short for a realistic scenario [6,28,51]. Another study used hand palms to measure $SPO_{2}$ by applying spatial averaging, obtaining R, G, B time series and applying CNN structure; however, using palms under a camera is not very practical as people in real life would be required to keep their hands still under a camera, something that is unrealistic in healthcare settings [27].

Previous studies have used a variety of equipment and set-ups to capture data from participants to obtain HR. Thermal [52,53,54], charge-coupled device (CCD) [55,56], other affordable web cameras or those built in laptops [19,22], Microsoft Kinect V2 [57], GoPro camera with drone [58], and smartphone [27] cameras have been previously used to obtain a person’s vitals. Each device has its own characteristics, including resolution, dimensions, processing power, data type collected, and cost. Low and high-resolution digital cameras and smartphone cameras capture R, G, and B channels, which have been used in most of the studies to obtain vital sign data, whereas a thermal camera allows for capturing the thermal data of a participant; however, expensive equipment such as thermal or CCD equipment would not make it practical or cost-effective to deploy within homes or in clinical settings. Smartphones need to be continuously held in the hand, which can be tiring and impractical for continuous vital sign monitoring.

Previous research data were gathered mostly in a controlled environment or lab setting [19,22,23,24,25,26,27,33,49,51]. Studies conducted restrict participants from micro facial gestures or physical movement of their arms or face rotations. The participants only moved slightly, which would create higher noise, and the system might not perform as expected in a real-life scenario. In a real-time situation, participants’ home environments may vary and different factors such as face rotation, facial expression, varied distance from the camera, illumination, beards, skin colours, etc., can be present, which can impact the system accuracy. Only a limited number of studies have discussed system accuracy on the basis of skin pigmentation; however, the RMSE is quite high [25] as shown in Table 1 and none have shown the system accuracy distribution with regards to makeup and a beard. In addition to that, no research discusses the impact of fps on the signal data as different hardware accessible to people might impact the system due to its RAM or other hardware capabilities. It is also unclear which signal processing techniques and algorithms perform well in a real-life scenario; therefore, our research focuses on creating, optimising, and validating an rPPG system in a real-life environment which studies the different factors described above.

## 3. Methodology and Participant Information

The study received ethical approval from the University of St Andrews (CS14639). The participant information sheet was presented, and consent was collected electronically using Qualtrics (online). Selection criteria included those who were able to consent for themselves and were aged 18 or above. Participants were informed that their taking part was voluntary and could withdraw before the data collection was completed. They were informed about the data collection and storage procedure before participating in the study. Participants’ personal information such as email and postal address were deleted permanently after they completed the research study and returned the equipment (where any equipment was sent to them). They were also informed that this was a scientific study and the technical researchers were not qualified medical professionals—it was mentioned in the participant information sheet and consent form that it is the participant’s responsibility to immediately contact emergency service or GP, in the case that the ground truth device detects an ‘abnormal’ heart rate or oxygenation level during the data acquisition (resting heart rate below 40 BPM or more than 130 BPM, or blood oxygenation level less than 90%). The study would also be terminated for that participant instantly. The participants took part in the study remotely from their homes (real-life environments) from different parts of the world in order to obtain the widest variety of environments and skin pigmentation.

A total of forty (40) participants took part in the study where remote measurements from the ARPOS system and a commercial pulse oximeter (ground truth) device were obtained. The majority of participants were from the UK, Pakistan, and a few from Malta. In order to take part in the research study, participants were required to have a Kinect V2, a commercial pulse oximeter device, and a Windows OS PC or an Xbox One. Participants within the UK who did not have access to a Kinect V2, its USB adaptor, and a commercial pulse oximeter device were sent these items via university arranged courier. All the equipment was sanitised with alcohol wipes before sending it to the participants. Participants within the UK were also sent a book voucher to thank them for their participation. Participants from outside the UK were selected on the basis that they had access to the required equipment. The protocol steps followed in the ARPOS research study are published and available at https://dx.doi.org/10.17504/protocols.io.n2bvj6zkxlk5/v1 (accessed on 27 June 2022). Detailed participant information relating to participants’ age, gender and country of residence distribution is shown in Table 3. Participants who conducted the research study in dim lights (or a dark room with curtains closed) were asked to repeat the study with appropriate lighting. The data acquired in dark light affected the fps and to study the impact of fps, participants were also asked to redo the study. Participants with makeup were also asked to repeat the study without the makeup. The data collected from participants before and after re-doing the study were both included in the data analysis process to validate the system for various conditions within participants’ home environments that are discussed in the results section. Participants in the study included people who were able to operate Windows computers, laptops, or Xbox One. They also had the capability to set up the equipment and were able to understand instructions in English. Participants who did not understand any information provided on the participant information sheet or consent form were contacted by the researcher. The researcher then explained and translated the information into languages, which included English, Urdu, and Sindhi.

**Figure 1:**
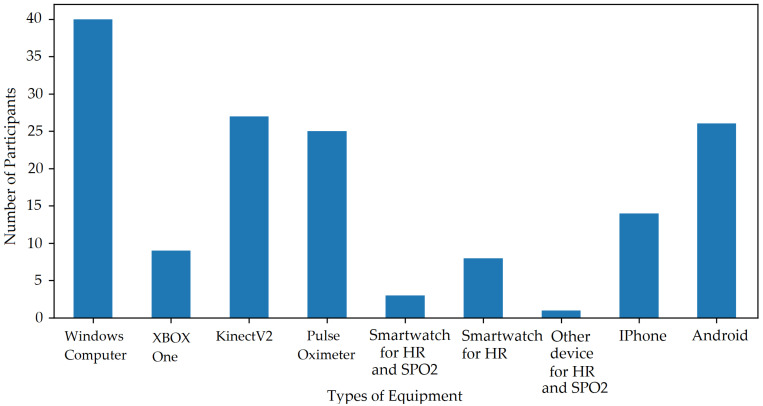
Participants Equipment distribution. Plot shows participants who had required equipment to take part in the study and remaining participants were sent the equipment if they resided within the UK.

Table 3 also shows the distribution of equipment used by the participants worldwide. The results in this research paper show data from participants with white and darker skin pigmentation. Asian participants with a pale skin colour were grouped with white skin participants’ data whereas the darker skin pigmentation group includes brown and dark skin colours. This was to study the effect of skin pigmentation for white and darker skin colour rather than the country of birth or ethnicity distribution. Figure 1 show different equipment owned and used by participants for the ARPOS research study.

The ARPOS study was designed to be conducted remotely and accommodated worldwide participation. To conduct the ARPOS research study remotely, a ground truth device to measure HR and $SPO_{2}$ was required for which a preliminary study was designed to select a commercial pulse oximeter device that produced vitals closer to a clinical pulse oximeter device as the clinical device is costly and fragile to post to multiple participants at a time. Three commercial pulse oximeter devices and NONIN 2120 clinical pulse oximeter (NONIN2120 (Clinical Pulse Oximeter), https://www.nonin.com/support/2120/ accessed on 27 June 2022) were used within the preliminary study. The three pulse oximeter devices were selected based on the criteria that they had bluetooth connectivity, easy user interaction, supported Android and iPhone, allowed smartphone to record screen, had good user reviews, and under a budget of 51.70 GBP (65 dollars). The commercial pulse oximeter devices included iHealth (iHealth Air, https://ihealthlabs.com/products/ihealth-air-pulse-oximeter accessed on 27 June 2022), Wellue (Wellue FS20F, https://getwellue.com/products/fs20f-bluetooth-finger-oximeter accessed on 27 June 2022), and Contec (Contec CMS50D-BT, https://bit.ly/3OKbSnd accessed on 27 June 2022) devices. The Wellue pulse oximeter was found to be the most suitable device, with an RMSE of 4.5 (mean value over resting and active states) for HR and 1.34 for $SPO_{2}$ compared against the NONIN 2120 clinical pulse oximeter device.

### 3.1. ARPOS Research Study Steps

The following steps were followed by participants to complete the research study. The experiment protocol for this research study has been made open-source and is available at [59]. Participants were encouraged to go through the guide before carrying out all these steps to ensure smooth execution. Participants were asked to repeat steps 6 to step 13 three times, meaning twice for a resting state and once after exercise. Resting state means when participants vitals were in a resting state, such as sitting on a chair without any exercise (to acquire their resting HR and $SPO_{2}$). The second resting state was added to further validate the system. After exercise is when a participant does some form of exercise (star jumps were recommended) for 60 s to increase their HR and immediately take their vitals from the ARPOS and ground truth system. Participants were asked to do the last round only when they were ready with the study setup and start the application as soon as possible after exercise so the system could acquire an active vital data reading.

**Step1:** Consenting to the Study

Participants were asked to sign up for the study using Qualtrics. A participant information sheet was also included on the same form which explained all the necessary information related to the research study. The researcher’s contact details were also provided along with a link to making complaints if they had any concerns regarding the research study.

**Step2:** Participant Id and Receiving Auto-Generated Emails

Once participants consented to the research study, an auto-generated email with the participant information sheet and a copy of the consent form including their participant Id (e.g., PIS-001) was sent to them. The email also provided a link to the research study website to follow the next steps of the research study. The researcher also received a copy of the participant’s sign up sheet. At this stage, the researcher would reach out to the participant to discuss study equipment postage if required; otherwise, ask them if they had any questions regarding the researcher’s study.

**Step3:** Equipment Postage to Participant

The researcher asked the participants about the best available date on which they could receive the equipment at their home address. Once the participants confirmed their postal addresses and dates, the researcher then arranged a courier service with the help of the University to post equipment to the participant.

**Step4:** ARPOS Study Setup

**Figure 2:**
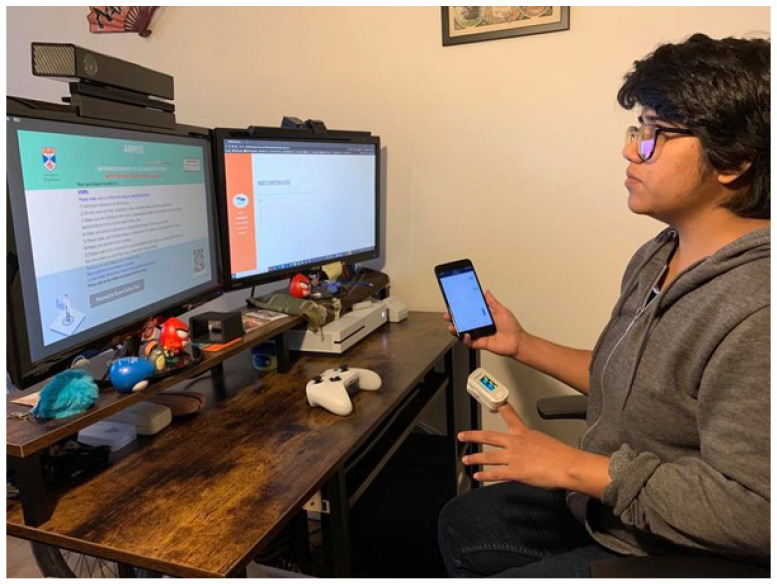
Final setup of the research study where the Wellue pulse oximeter and the ARPOS system acquire data simultaneously [59].

Participants were asked to search and download the ARPOS application from the Microsoft store to their device. Participants were asked to set up the research equipment before starting the application where Kinect V2 was to be placed in an appropriate position, and the person’s face was in front of the camera with appropriate light within a room and without a face covering. Once the ARPOS setup was completed, they were asked to mount the pulse oximeter clip on a finger and pair their smartphone with the device as per the document guide provided. Final study setup is shown in Figure 2.

#### 3.1.1. Step 6: Executing the ARPOS Study

To log in to the app, participants were required to have a PI number. This participant Id was activated by the researcher on the server-side once the equipment was received by the participant or once they confirmed their participation in the research study. If the participant’s Id was not activated, the application would give the message, “This participant number is not correct. Please re-enter or contact the researcher if you are not sure”. Once the participants had completed the research study, they could no longer log in to the application. This was to avoid overwriting data once it had been received on the server. The message displayed was, “You have already completed the study. Thank you very much for your participation”. Once the participants were ready and facing Kinect and screen recording for the pulse oximeter data had initiated, they could start the data acquisition by clicking the “Start capturing face data” button.

#### 3.1.2. Step 7: Processing and Sending Data to the Server

Once the application completed data acquisition, it stored participants image frames user’s disk, after which it would process the data locally and update the user to not close the application. The system cropped face data from colour, IR, and depth images, and discarded rest of the background. The depth data were then used to obtain distance from participants face to the sensor. The colour face coordinates were mapped to the IR image to obtain IR data from 16-bit grayscale image. When the data were ready to be sent to the server, they provided the participant the option to either share using their OneDrive (in case the internet connectivity was not stable) or send directly through the application. Secure sockets layer (SSL) was installed on the API link and the data were serialised in binary format to secure data transfer from the client to the server side. After the data were transferred on the server, they were deleted from the user’s disk to free the up the space. Participants then repacked the equipment and sent it back to the researcher.

## 4. ARPOS System

The ARPOS system created and designed by the authors of this paper measures vitals including HR and $SPO_{2}$ remotely. The main focus of this research was to find an alternative way to help monitor the physical health of people in a remote contact-free manner while catering to their privacy concerns; therefore, the system does not store any image or video data of participants being monitored. For the purpose of validating and optimising the system, face data from participants in colour, IR, and depth were acquired and stored on the University of St Andrews server. Obtaining HR and $SPO_{2}$ vitals is possible using a variety of optical wavelength bands (colours) in the visible and the infrared regions, the different optical absorption properties of oxy-haemoglobin and deoxy-haemoglobin may be used to calculate the blood oxygenation level.

**Figure 3:**
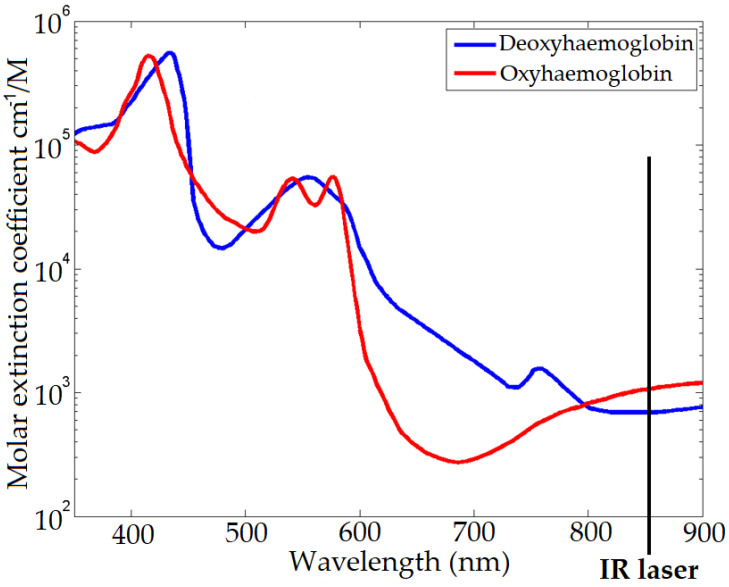
The optical extinction spectra of oxy-haemoglobin (red line) and deoxy-haemoglobin (blue line) within blood. Fig generated using data by Prahl [60].

**Figure 4:**
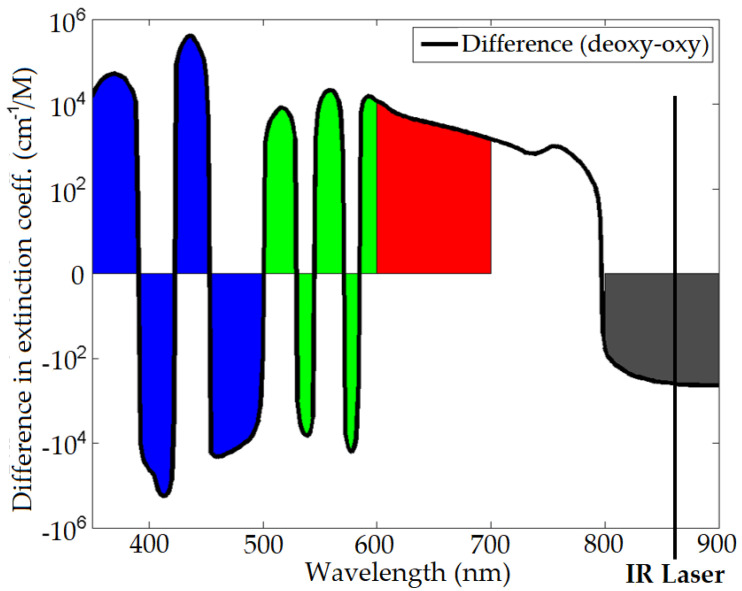
The spectral extinction coefficient differences between deoxy-haemoglobin and oxy-haemoglobin (deoxy minus oxy, shown with a black line). Shaded areas correspond to the spectral regions of colour camera channels red, green, and blue; the grey shaded area is the near infrared (IR) spectral region above 800nm, which IR cameras, such the Microsoft Kinect games console camera, are able to detect. Fig generated using data by Prahl [60].

The extinction spectra of oxy-haemoglobin and deoxy-haemoglobin is shown in Figure 3, which is a measure of how much light is reduced due to absorption and scattering at each wavelength, and the spectral differences between them for the imaging colour bands. A ratiometric measurement, found by dividing the measured pixel intensity between two different spectral regions, e.g., red (600–700 nm) and infrared (between 800–900 nm), can provide a measure of the oxygenation level of the blood as shown in Figure 4. The Kinect V2 IR laser wavelength was measured using a spectrometer (Ocean Optics Flame-S-VIS-NIR), it was found that the laser wavelength is 860 nm. Areas of exposed skin such as the face can be recorded to measure the surface changes. Although not visible to the naked eye, as the heart beats, the skin is flushed darker and then brighter; this modulation in intensity means the heart rate may be calculated by recording the image pixel intensity at the skin surface over time. Figure 3 and Figure 4 are generated using data by Prahl [60].

**Figure 5:**
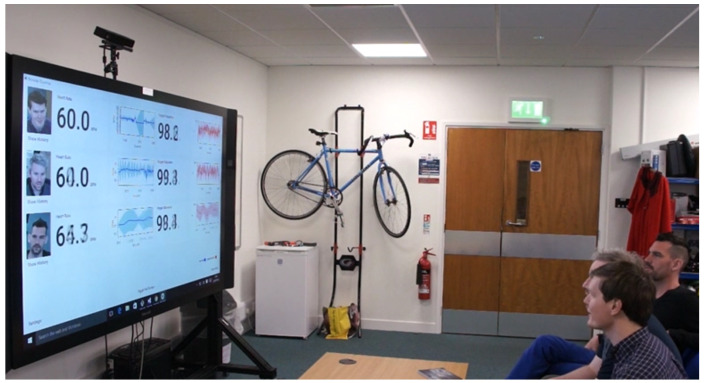
ARPOS live application demo image. The image shows peoples faces are identified on the left (monitor screen) where the heart rate is the blue line and blood oxygenation level is the red line plotted on the screen where the shaded regions represents the potential error.

**Figure 6:**
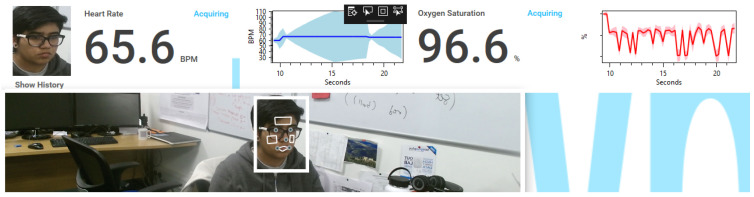
Sample reading from the system. The image showing the regions of interest (forehead, cheeks, and lips) automatically extracted from the face.

**Figure 7:**
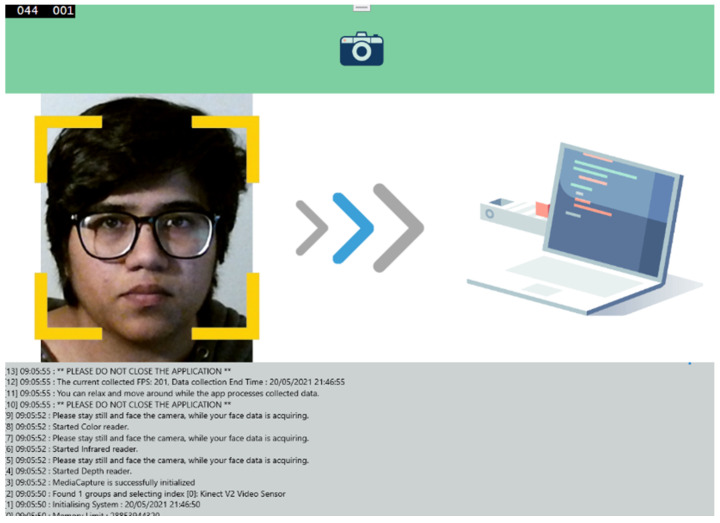
Screenshot of data acquisition study. A screenshot of what participants see during the data acquisition study showing that their face has been identified correctly and showing a log to update the user about the next steps.

Compared to existing systems, the novel aspect of this study using the ARPOS system is that it has been tested in various environments and deployed and operated by users within their homes worldwide (specifically in Europe and Pakistan). The ARPOS system has been validated for different factors such as different skin pigmentation, real-life environments, various home illuminations, genders, and various other factors such as beard, makeup, and glasses. Currently, the real-time system can measure up to six peoples’ vitals up to a distance of 4.5 m from the camera. The system uses affordable off the shelf hardware that is easy to setup. Kinect V1 and V2 can capture colour (R, G, B), infrared, and depth data of a participant. Kinect V2 colour resolution is (1920 × 1080) comparatively higher than Kinect V1 (640 × 480). Kinect V1 for IR uses structured light, while Kinect V2 uses a time of flight (ToF) where the light source (photons) are used to measure distance from the object [61]. In the case of rPPG, it is essential to have ToF for a realistic scenario as the distance measurement is dynamic and has better coverage for wider interval. To give an indication of price, the Kinect V2 camera is currently available for prices as low as 25 GBP (Kinect V2 Price, https://uk.webuy.com/product-detail/?id=sxb1offkin2&sku=sxb1offkin2, accessed on 27 June 2022). A demo image of real-time ARPOS system is shown in Figure 5. Sample reading from the system user interface (UI) is illustrated in Figure 6. An output image from UWP data acquisition system is shown in Figure 7.

The ARPOS system for data acquisition was designed in Universal Windows Platform (UWP) and published on the Microsoft Store. The system required at least a RAM of 5 GB and successfully operated on all participants’ devices. The system was designed to collect face data of individual participants to validate and improve the ARPOS system, which is then incorporated into the real-time application. Once the ARPOS data acquisition application was downloaded and initiated on participant’s devices, they mounted a ground truth device (Wellue pulse oximeter clip) on their finger and connected it to their smartphone, which the ARPOS system was validated against.

**Figure 8:**
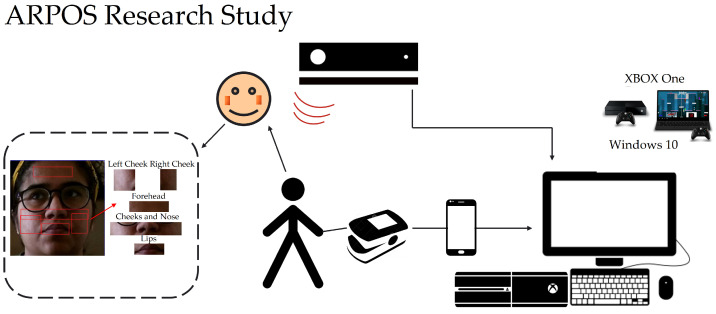
Illustration of data acquisition study design. Illustration showing the design of how the data were acquired in the study.

Figure 8 illustrates the data being recorded from the ground truth device and the ARPOS system simultaneously. Participants’ Id was activated once the equipment was sent to the participants within the UK or once they confirmed their participation. The active participant Id allowed logging in to the application; otherwise, they would be given a message to contact the researcher. In case the participants had completed the study, the participant Id was automatically deactivated. The server API and database was hosted within the University Server. The content was serialised and posted using a URL with an SSL certificate installed to secure data passage from client-side to server-side. MS SQL database was used to hold processed vital signs for different processing techniques.

The server held data of each participant by their participant number (for example, PI001). The API then deserialises the data objects to respective images and participant information such as Colour.png files, IR.png files, participantInformation.txt, which holds distance from the camera, frame timestamp information for colour, IR, and depth, along with a start and end timestamps. Post-processing on face data was performed in python to acquire HR and $SPO_{2}$. Participants conducted the research study for two resting states (Resting1 and Resting2) and one active state. The data were acquired for a total time of 65 s for each vital state (e.g., resting or active); however, only 60 s of data were processed to optimise and validate the system. The first and last few seconds were removed due to very low fps (as low as 3fps), which would not provide enough signal data to obtain vitals for that second. The extra few seconds were added to the data acquisition program to allow the participant’s device RAM to load the program and initiate threads required to capture the face data.

### 4.1. ARPOS System Process

**Figure 9:**
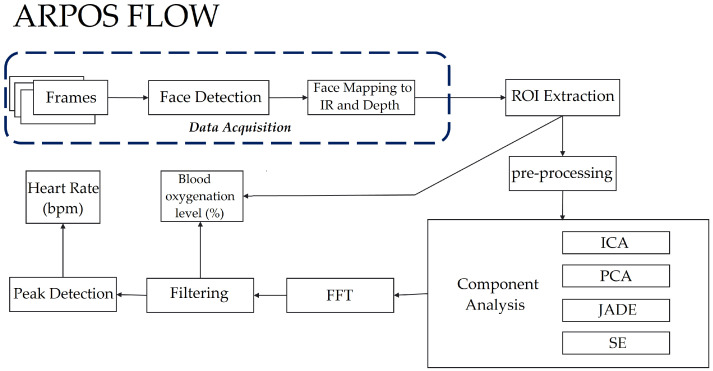
ARPOS Data Processing Flow Diagram. Showing the acquisition and the analysis for calculating heart rate and blood oxygenation level from obtained image data.

The ARPOS data acquisition and post processing to obtain vital signs is shown in Figure 9. All the code used to process the data collected from participants is available on GitHub at https://github.com/PirehP/ARPOSpublic accessed on 27 June 2022. Further, the corresponding anonymous extracted regions of interest data that were used are available at https://doi.org/10.5281/zenodo.6522389 (accessed on 27 June 2022). This is to allow reproducible research and allow future researchers to build on what we have achieved. The description of how this method works is also explained below:

#### 4.1.1. Data Acquisition

**Figure 10:**
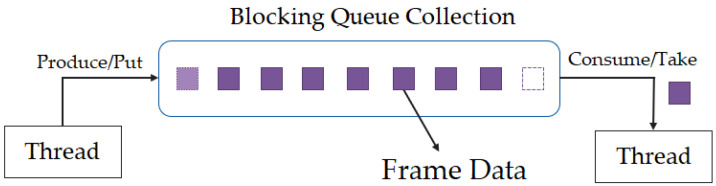
Blocking Queue Collection Concept. The image shows thread that puts the frame data in a blocking queue collection and another thread takes and processes it from the queue (extracts face data and writes it to the disk).

The system first acquires colour, IR, and depth frame data of participants, which is pushed into the blocking collection queue. Blocking collection is a class that holds thread-safe collection and allows the implementation of a producer–consumer pattern (BlockingCollection, https://docs.microsoft.com/en-us/dotnet/api/system.collections.concurrent.blockingcollection-1?view=net-6.0 accessed on 27 June 2022). In this case, the blocking collection holds a collection of frame data object consisting of frame information such as colour, IR, and depth frames with time stamp in a queue. Blocking collection allows a thread-safe concurrent addition and removal of frame data to the queue from numerous threads on a device enabling a lossless and low RAM data acquisition system. This concept is demonstrated in Figure 10. The face is identified from the entire frame where only the colour face of the participant is cropped with its co-ordinates. Since the IR frame has smaller dimensions but the participant is in the same location as in the colour frames, this can be identified by locating the position of participant using colour co-ordinates. This can be performed by identifying the width and height ratio where the original height and width for colour (1080 × 1920) can be divided by IR height and width (424 × 512). Once the width and height ratios are obtained, the co-ordinates of colour x, y, w, and h can be divided by these ratios, respectively, to obtain the new co-ordinates.

Similarly, the depth image is mapped to colour coordinates. IR and depth images are stored in 16 bits per pixel (as obtained from frames to retain the quality), whereas colour data are in BGRA8 (blue, green, red, and alpha with 8bits per channel) format. To obtain distance from depth data, first, the face is extracted using a similar technique to map colour coordinates to the depth image and then the centre of the image is obtained in meters by multiplying it by 0.001 as the distance obtained is in mm(millimetres) and depthWidth is 512. The depth data are copied using System.Buffer.BlockCopy, whereas IR pixels are calculated by creating a byte array with length = width × height × blockSize (2). The blockSize is two (2) as the IR image is 16 bits per pixel, so 16/8 = 2 where 8 bits exist in a byte. IR face is copied by copying each pixel in those coordinates to an empty byte array of calculated length. Face bounds for colour, IR, and depth were proportionately increased by a factor of 0.5 to 0.9 to capture the entire face in all frames without removing any part of the face.

#### 4.1.2. ROI Extraction

**Figure 11:**
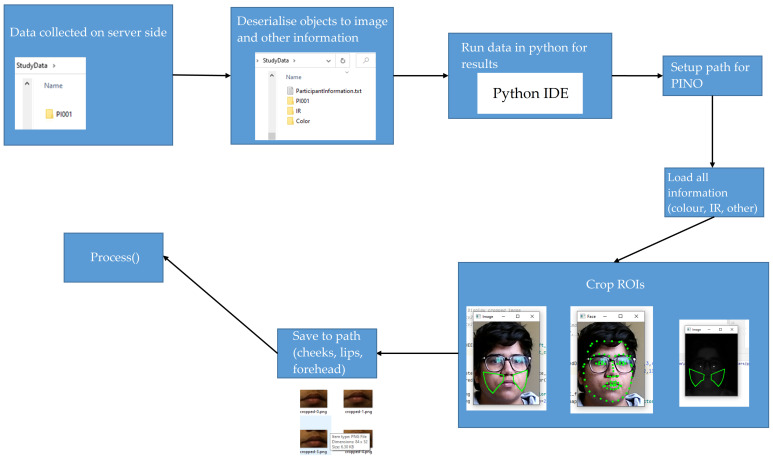
Flow chart showing post processing where ROI are extracted using Dlib in python from participants’ faces.

**Figure 12:**
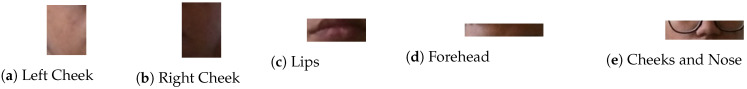
Colour ROI obtained from a participant’s face.

**Figure 13:**
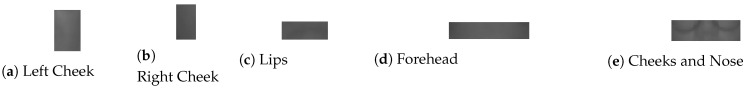
IR ROI obtained from a participant’s face. Images have been modified for clarity purposes.

The next step, as shown in Figure 9, was to extract ROIs from the face in colour and IR. The server stored the data of each participant by their participant number (for example, PI001). The API deserialised the data objects to respective images and participant information such as Colour.png files, IR.png files, participantInformation.txt. participantInformation.txt file holds distance from the camera, frame timestamp information for colour, IR, and depth, along with a start and end timestamps as shown in Figure 11. The ROIs extracted from colour and IR included right and left cheeks, right–left cheeks with the nose (combined), lips, and forehead. Initially, four ROIs (excluding combined cheeks with nose) were used; However, since participants were moving in front of the camera, another region from the centre of the face would be useful to add and to increase the likelihood of obtaining the best SNR. SNR was calculated for ROI with a larger and smaller (pixel) size. Figure 12 and Figure 13 show a visual example of ROIs extracted from a participant.

Dlib [62] with 81 face points was used to extract ROIs. IR image was enlarged to allow Dlib to easily identify ROIs and then apply a similar technique of mapping ROI points to original IR to extract identified locations. Spatial pooling was applied to average noise for pixel data from obtained ROIs. Grey channel was obtained by averaging R, G, and B values as a (64 bit) floating point value enabling a larger dynamic range for this single channel. Spatially pooled and processed data with a timestamp for each channel type, face distance for each second, fps for each second, vital ground truth was stored in binary ROIStore object form on the disk. This reduced load time and processing time for all participant’s data. The data could be extracted by providing a key related to the region, for example, ROIStore[region] = GlobalData().

#### 4.1.3. Pre-Processing

After spatial pooling, the raw signal data for all channels obtained were normalised as it may still contain some uncertain variation. The signal obtained was also interpolated, which means increasing the sampling rate. Each raw signal channel data were detrended to remove any unwanted trends present. If the fps was less than 15, additional upsampling was performed, where the signal was upsampled by a factor of 2, and this method uses FFT to perform upsampling (Resampling, https://docs.scipy.org/doc/scipy/reference/generated/scipy.signal.resample.html accessed on 27 June 2022).

**Normalise Signal:** [63]

(1) $$Signal_{channeltype}=\frac{Signal_{channeltype}}{np.linalg.norm(Signal_{channeltype})}$$

where channel type is R, G, B, Gy and IR.

**Interpolation:** The signal obtained is interpolated by estimating the linespace using length of the signal data and its maximum time maximumTime=Len_S/FPS_channel and then apply interpolation as mentioned below:

(2) $$nterpolatedData=np.interp(eventimes,timesteps,Signal_{channeltype})$$

where eventimes is obtained by numpy method that returns values spaced evenly with respect to interval eventimes = np.linspace(timesteps[0], timesteps[−1], len(timesteps)).

**Hamming Signal:** Numpy python library is used as shown below [64]:

(3) $$Signal_{interpolatedchannel}=np.hamming\left(L\right)*interpolatedData$$

**Median Filter:** The library used is scipy.signal.medfilt in python as shown below:

(4) $$Signal=signal.medfilt(Signal_{interpolatedchannel})$$

**Detrend:** The library used is scipy.signal.detrend in python as shown below:

(5) $$Detrended_{channeltype}=signal.detrend\left(Signal_{channeltype}\right)$$

where channel type is R, G, B, Gy, and IR.

**UpSample:** Scipy library was used to up sample the signal in python as shown below:

(6) $$signal.resample(Signal_{channeltype},len\left(Signal_{channeltype}\right)*2)$$

where channel type is R, G, B, Gy, and IR.

#### 4.1.4. Noise Reduction Analysis

The signal data obtained after pre-processing may contain noise and an undesired mixture of signals. To separate noise and extract the source of signal, noise reduction algorithms were applied, which included FastICA [65], PCA [66], JADE (JadeR, https://github.com/gbeckers/jadeR accessed on 27 June 2022), and SE [67]. Some of the algorithms were implemented with different pairings; for example, applying PCA and then applying ICA. This was chosen as PCA can be used to whiten data and help reduce noise further when independent components are extracted from ICA. The signal data were also processed with no noise reduction algorithm (in this paper we have named it as ‘None’ on the plots) to compare its results with that of different algorithms. Along with that, the signal data were also passed multiple times through ICA to see if this would help improve extracting independent components. It was noticed that applying ICA more than three times has no effect; therefore, we only applied ICA once and then three times on the signal data. The various noise reduction algorithms were applied with different pre-processing techniques as mentioned in Table 4. Pre-processing technique 3, 4, and 5 did not extract signal data that could provide enough information to obtain vitals and therefore no more information is shown on these for conciseness.

#### 4.1.5. Fast Fourier Transform (FFT)

A FFT converts signal data from their original domain to the frequency domain and vice versa; therefore, a FFT was applied to convert the signal data from their original domain (number of samples across time) to the frequency domain.

#### 4.1.6. Frequency Filtering

A bandpass filter was applied to remove unwanted features from the signal data where the lowest frequency was 0.66 Hz (0.66 × 60 = 39 BPM) and for removing frequency above was 3.33 Hz (3.33 × 60 = 200 BPM). This is because generally a persons HR is within this range (39 BPM TO 200 BPM), so unwanted signals were removed below and above these, respectively.

#### 4.1.7. Calculating HR (BPM) and $SPO_{2}$ (%)

To calculate HR, the highest peak in the frequency domain was multiplied by 60 (frequency x 60) to convert it into BPM. The highest peak was identified by first identifying peaks (Scipy findpeaks, https://docs.scipy.org/doc/scipy/reference/generated/scipy.signal.find_peaks.html accessed on 27 June 2022) from the signal on which FFT was applied and then selecting maximum peak and its frequency. The system also calculated the error rate for HR and $SPO_{2}$ to show the reliability of that value. All channels were observed for peak value, and HR and $SPO_{2}$ value was selected, which had the best signal-to-noise ratio (SNR) value. The threshold for SNR was set to 5.0. This was applied for each region (lip, cheeks, etc.) and the best SNR among all channels and regions was chosen for vital values. The SNR formula used in the ARPOS system is shown below:

(7) $$Snr_{channel}=\frac{Frequency\_PeakValue_{channel}}{numpy.average(fft\_Signal)}$$

For $SPO_{2}$, the system applied inverse fast Fourier transform (iFFT) to the filtered grey signal, after which peaks were identified. For each peak index, raw IR, red signal (with only spatial pooling applied to it), and distance values were identified. Values for Hb and deoxyHb were selected from [60] for IR wavelength band 860 nm and for red, the average value from 600–700 nm was selected. Equation for calculating oxygenation levels is given below, where 52 is the IR/R scaling factor. C is a constant value deducted (for example in this case 6) from the final oxygen level obtained as a constant offset value occurred for all participants data:

(8) $$irToRedRatio=\left(\frac{redoxymean}{iroxymean}\right)\times \frac{\left((irValuex(distValue)/redValue)\right)}{52}$$

(9) $$oxygenLevel=100\times \frac{\left(reddeoxymean-(irToRedRatioxirdeoxymean)\right)}{\left(iroxymean+reddeoxymean-irdeoxymean-redoxymean\right)}-C$$

Finally, a reliability check was also applied to ensure the calculated vital signs (HR and $SPO_{2}$) for the next window second was a reliable reading. For example, if a participant’s HR is stable at 60 BPM, it would not raise to 144 BPM for just one second. A vital value with SNR below a certain threshold (in the case of this research study 5.0) would be rejected and the previous vital sign value would be selected. In addition to that a deviation acceptance factor was used to check if the newly measured vital value deviates from the previous vital value by a certain percentage threshold (in this research study, the factor was 0.18 for HR and $SPO_{2}$). In the case that the vital sign deviation was much higher than this factor, the previous vital value would be selected and the newly measured value would be rejected. This is performed as the vital values can only increase or decrease by a certain percentage as the change should be gradual.

#### 4.1.8. Window Comparison

**Figure 14:**
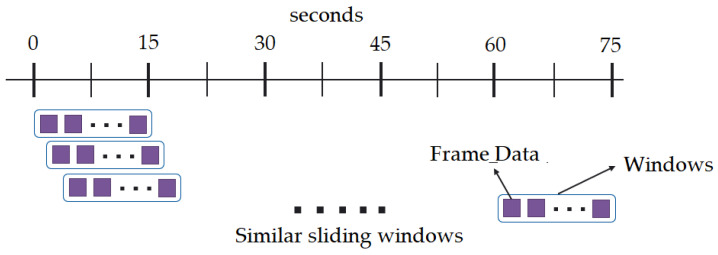
Sliding Windows Concept. The scale shown in the image represents number of seconds and grouped frame data are held inside a window of a particular size (which can be 4, 10, or 15). This window slides by 1 s and passes the data from that time frame window to the ARPOS system to measure vital for that specific window.

**Figure 15:**
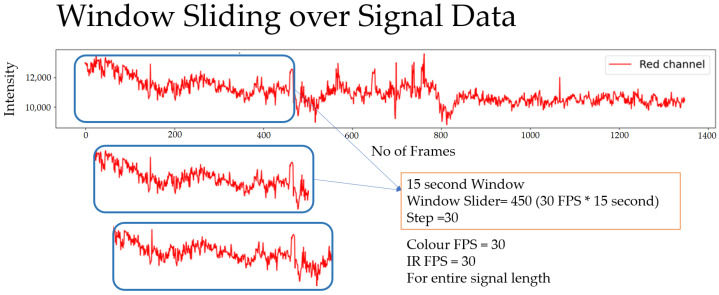
Window Sliding over signal data. A participants data are shown as an example where window of 15 s selects data for colour and IR and slides by 1 s.

The ARPOS data and ground truth data obtained were compared in form of window size of 15 s with a sliding step of 1 s. The sliding window concept is illustrated in Figure 14 and a visual example using signal data from a participant is shown in Figure 15 where a specific number of seconds is selected for a window and is then slid by 1 s to compare it with its counterpart, i.e., ground truth.

## 5. Results and Discussion

**Figure 16:**
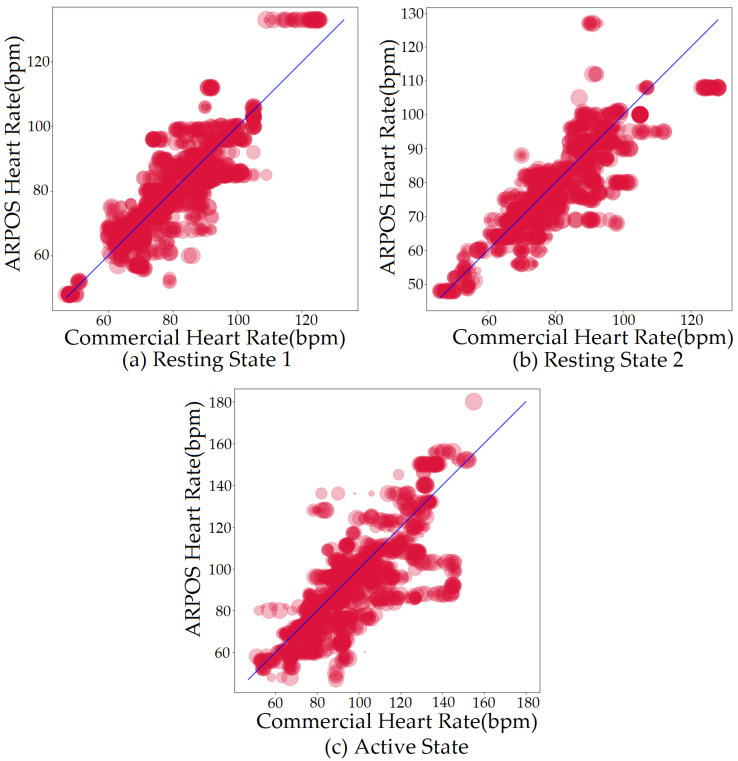
ARPOS system and ground truth comparison for all (40) participants for HR (BPM) for resting and active states. For resting state, plot selected with lowest RMSE value using FastICA with pre-processing technique 6 for fps greater than 15 and pre-processing technique 7 for fps lower than 15, which is also detailed in Section 10 and Table 4. For active state, plot selected with lowest RMSE value using PCAICA and similar pre-processing techniques as resting state. The data from each participant are obtained and shown for 60 s where the larger size and darker colour of sample points indicates the overlapping data on that point.

The results from the ARPOS system are presented in this section. Figure 16 shows comparison between the ARPOS system and the ground truth device for HR for resting and active states. Table 5 shows the mean vital values (HR and $SPO_{2}$) obtained for each state across all participants.

There are several reasons why on the rare occasion the values of the commercial pulse oximeter and the ARPOS system are dramatically different (outliners shown in the Figure 16). These reasons may include either issues with the commercial pulse oximeter or issues discussed below for skin pigmentation (Section 5.2), make up (Section 8), and beard (Section 9) on the obtained vitals from the ARPOS system.

### 5.1. Evaluation Measures

Evaluation measures presented in the results are defined below:

**Mean Difference for vital signs:** The difference for vital values is obtained by subtracting ARPOS obtained values from ground truth vital values where vital means HR and $SPO_{2}$. $VitalDifference=GroundTruth_{vital}-ARPOS_{vital}$ The mean difference for HR and $SPO_{2}$ is measured by:

(10) $$\bar{mean}=\frac{1}{N}\sum_{i=1}^{n}\left(VitalDifference_{i}\right)$$

where *N* is the size of vital difference dataset. The mean absolute difference for HR and $SPO_{2}$ is measured by:

(11) $$|\bar{mean}|=\frac{1}{N}\sum_{i=1}^{n}\left(|VitalDifference_{i}|\right)$$

where *N* is the size of vital difference dataset.

**Standard Deviation Error (σ SE):** The standard deviation (SE) is a calculation of the variation of the vital values. A low SD value suggests that the values are close to the HR and $SPO_{2}$ mean, while a higher value means that the HR and $SPO_{2}$ values are spread out over a broader spectrum.

(12) $$\sigma =\sqrt{\frac{\sum {\left(eachVitalValue_{j}-VitalMean\right)}^{2}}{Sizeofvitaldataset}}$$

**Root Mean Square Error:** The root mean square error (RMSE) is calculated by taking the difference between the ARPOS observed vital values and the ground truth vital values.

(13) $$\sqrt{\frac{\sum_{i=1}^{n}{\left(Actual_{j}-Observed_{j}\right)}^{2}}{Sizeofvitaldataset}}$$

**Correlation coefficient (r):** Pearson’s r correlation coefficient is a measurement of linear correlation between two the ARPOS obtained vital values and the ground truth vital values. r value closer to one (1) or higher value indicates a higher correlation between the data sets and the opposite for a lower r-value.

Participants data include variations in both illumination and movement as long as it is in front of the camera. Some participants were talking, laughing, and very few moving to some degree (approximately 45 degrees). During the study, there was only one participant internationally (participant from outside the UK) who had an abnormal vital reading and did not have a steady hand. For this reason, the study was terminated for that participant, and they were asked to seek help from their family members and contact a doctor. The data collected consist of variable fps ranging from 3 to 30 (depending on participants hardware). ROIs include left and right cheek, cheeks and nose, lips, and forehead. Microsoft (MS) face tracking in UWP was used instead of Kinect V2 SDK as its features are not easily accessible in UWP for XBOX One. This would enable the system in the future to not be camera dependent, meaning any camera can be integrated with the system that acquires colour, IR, and depth data as computer vision and face mapping techniques have been used to locate face data.

**Figure 17:**
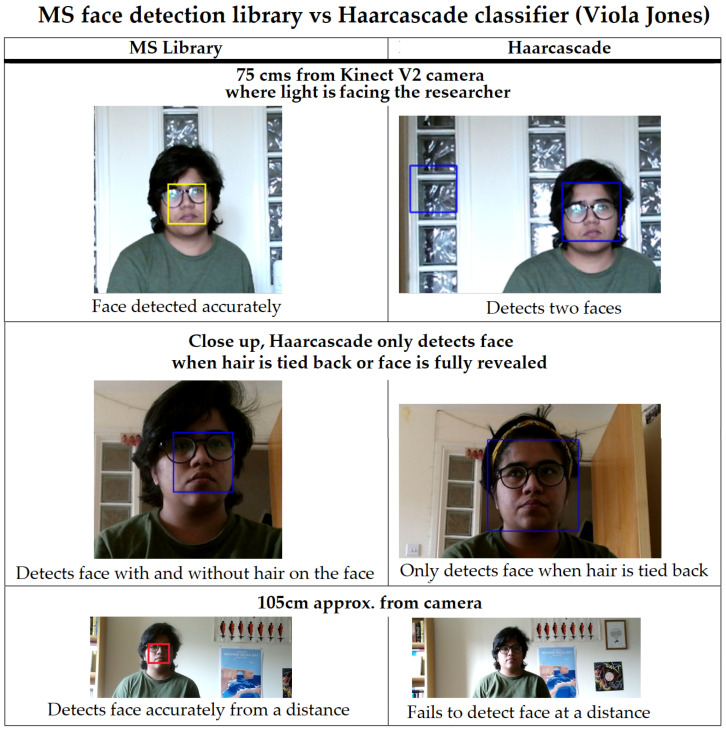
Microsoft (MS) face detection library vs. Haarcascade classifier(Viola Jones). Microsoft face detection library detects faces in different cases compared to Haarcascade.

ROIs were identified using Dlib [62] python face tracking. Harcascade (Viola-Jones) [68] and MS face library (Face Detector Class) [69] were compared to find the library that produced optimum results (detecting faces) in a home setting. Viola-Jones was selected as it was most commonly used in previous research for identifying faces [6,58,70,71]; however, Viola-Jones did not perform well (failed test cases) compared to MS face library as shown in comparison in Figure 17. Face detection from the full frame was performed for every 7th frame arriving to the frame reader. This was to allow participants with low RAM (PC’s or XBOX One) to take part in the research study as face detection for every frame would be computationally expensive (requiring RAM > 5 GB and CPU processing power of > 2 gigahertz (GHz)). Face detection for every frame is possible if the system is deployed on a device that has a higher RAM capacity of more than 8 GB and CPU of 2.90 GHz. In any case, this does not impact the quality of data acquired as only face dimensions of the last frame are mapped onto the new one and retaken for every 7th new frame arriving where the fps of the camera should be 30. Full frame dimensions obtained using Kinect for colour was 1920 × 1080 and 512 × 424 for IR and depth. Face frame dimensions varied depending on face location in the frame and distance (depth data) from the camera. Channels used in the system included red (R), green (G), blue (B), gray (Gy), and infrared (IR).

Previous systems that acquire HR have been listed in Table 1 for HR and Table 2 for $SPO_{2}$. The ARPOS system accuracy measures for HR and $SPO_{2}$ are shown below in Table 6 and Table 7. The ARPOS system accuracy measures for HR are comparable to the recent study [26] mentioned in previous work, which also has same number of participants; however, the study is based in a controlled environment, does not provide accuracy measures based on skin pigmentation and other factors common in real-life scenarios, for example, beard and makeup, and only measure HR. The rest of the previous studies have low participation, which ranges from 4 to 27. The lab-based studies or controlled environments with low participation and diversity do not stress test a system that would cause a system to fail in a realistic scenario. Table 2 for $SPO_{2}$ shows the error rate increasing for higher participant of 14 compared to that of low participation of 4.

Compared to the previous systems ARPOS system has been validated for various factors that occur in a real-life scenario, such as different skin pigmentations, ethnicities, various home illuminations, makeup, beard, computational expense, and fps impact on the results. In addition to that, the system proves its ease of use and affordability as participants deployed the systems and setup within their homes from different socio-economic and cultural backgrounds.

**Figure 18:**
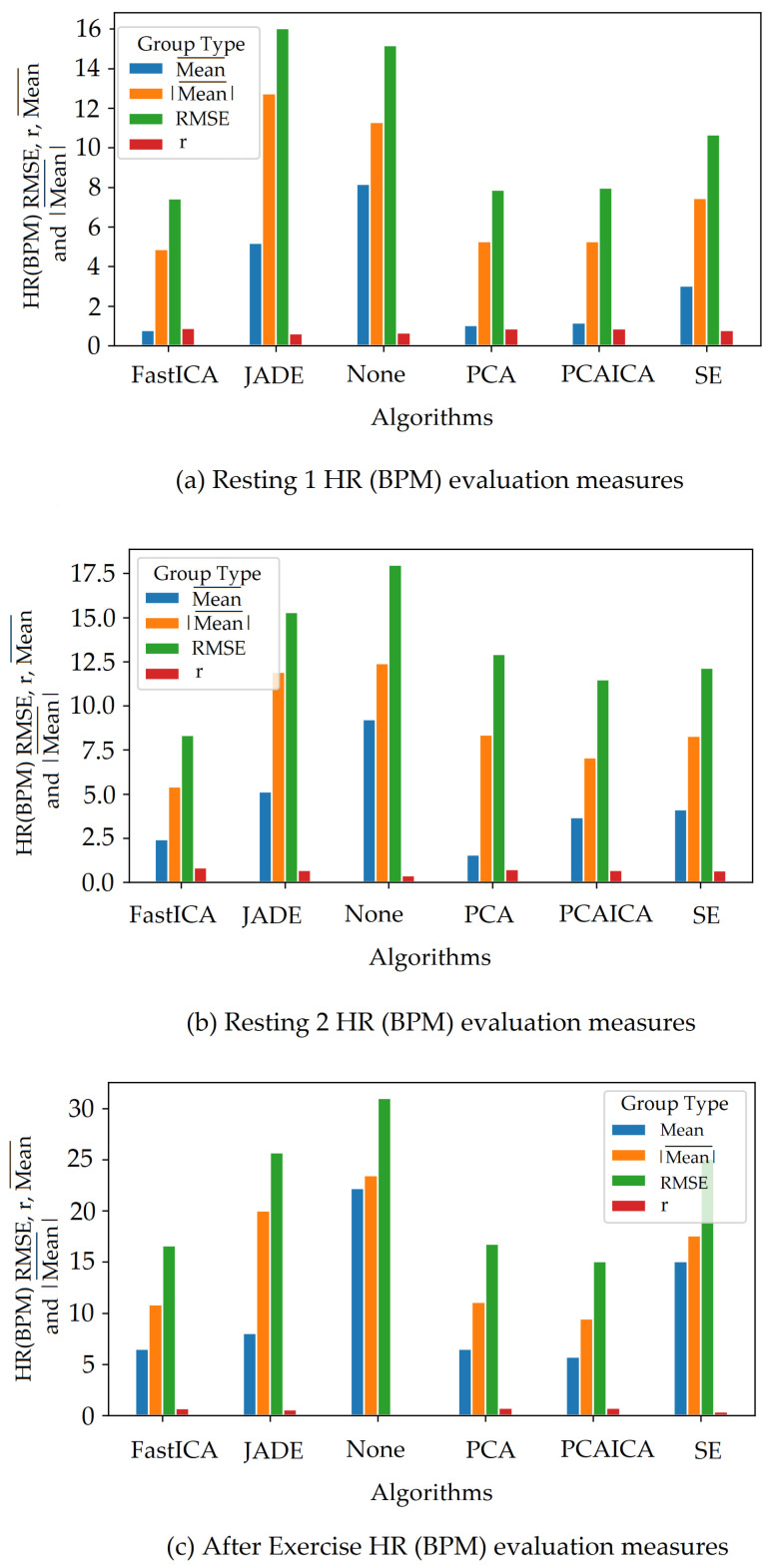
HR (BPM) RMSE values for all participants for resting and active states. The plots show Mean ($\bar{mean}$), MeanAbs (mean absolute error $|\bar{mean}|$), RMSE, and r values for different noise reduction algorithms where FastICA has best (lowest errors values with highest r correlation values) values compared to rest of the algorithms. The signal data were also processed with no noise reduction algorithm, which is represented by ’None’ on the plot and compared with rest of the noise reduction algorithms.

Spectral embedding (SE) [67] is a non-linear dimensionality reduction algorithm (LE) available in python (Spectral embedding, https://scikit-learn.org/stable/modules/generated/sklearn.manifold.SpectralEmbedding.html accessed on 27 June 2022). Previous research applied LE [49] to obtain HR and revealed a mean difference of −0.3 BPM in comparison to ICA, which was shown to be −0.29 BPM. The research did not state RMSE, r correlation, or mean absolute difference values. This ARPOS study has found that SE does not necessarily perform the best for a data set collected from a real-life scenario as shown in Figure 18 where FastICA has the lowest RMSE and a higher r correlation value (also shown in Table 6).

### 5.2. Skin Pigmentation

**Figure 19:**
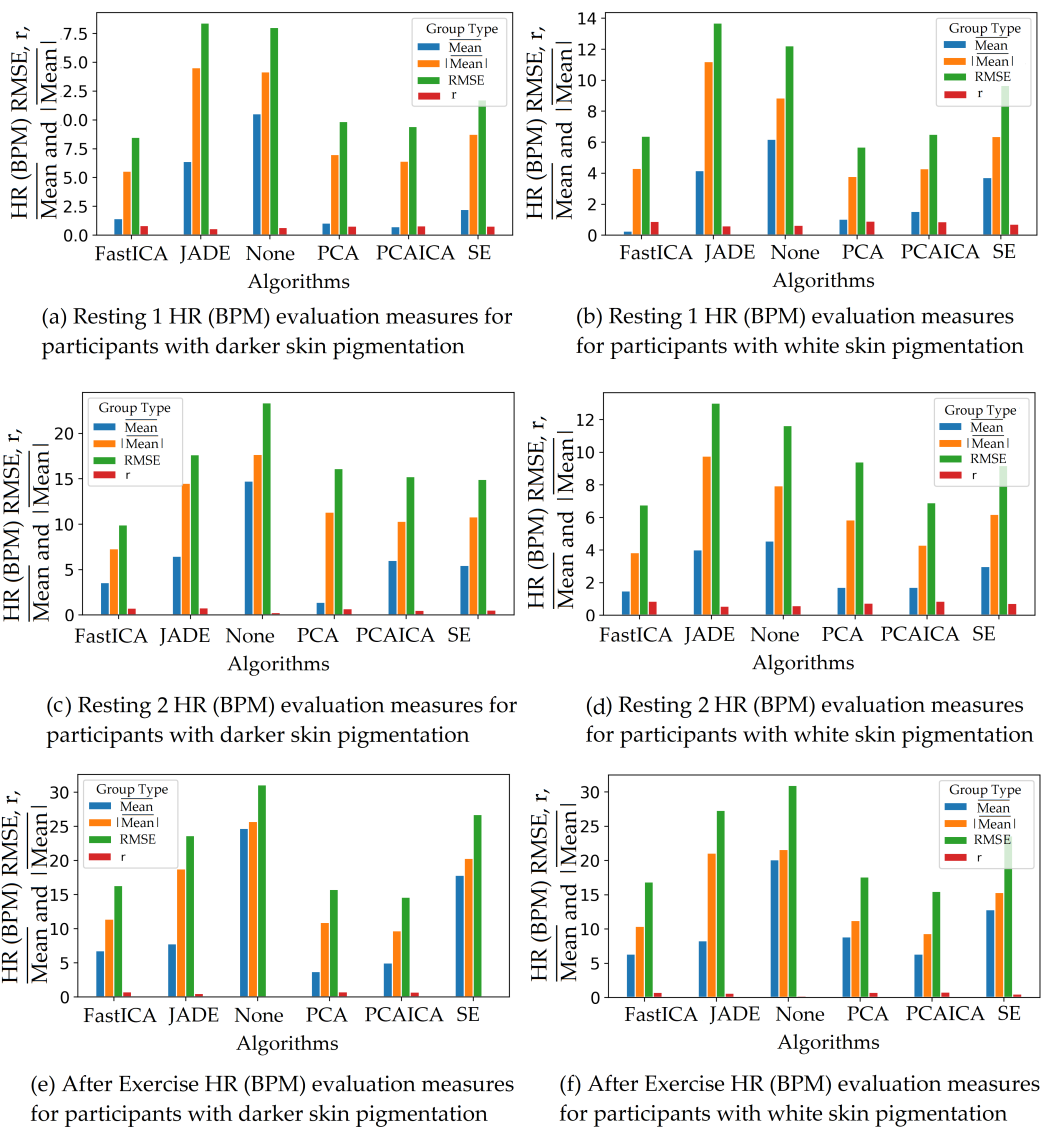
HR (BPM) evaluation measures for participants with darker (left side) and white(right side) skin pigmentation. Plot shows $Mean\to MeanError\left(\bar{mean}\right),MeanAbs\to MeanAbsoluteError(|\bar{mean}\left|\right)$, RMSE, and r correlation for resting and active states measured over participants with darker and white skin pigmentation.

**Figure 20:**
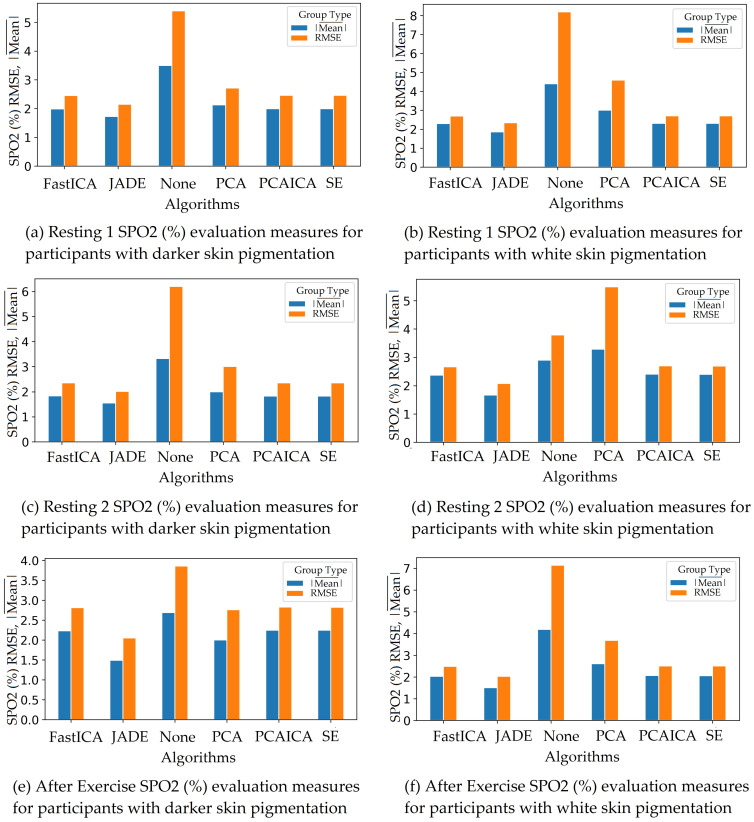
$SPO_{2}$ (%) evaluation measures for participants with darker (left side) and white (right side) skin pigmentation. $SPO_{2}\left(\%\right)$ evaluation measures ($MeanAbs\to MeanAbsoluteError(|\bar{mean}|)$, RMSE) for resting and active states measured over all participants.

**Figure 21:**
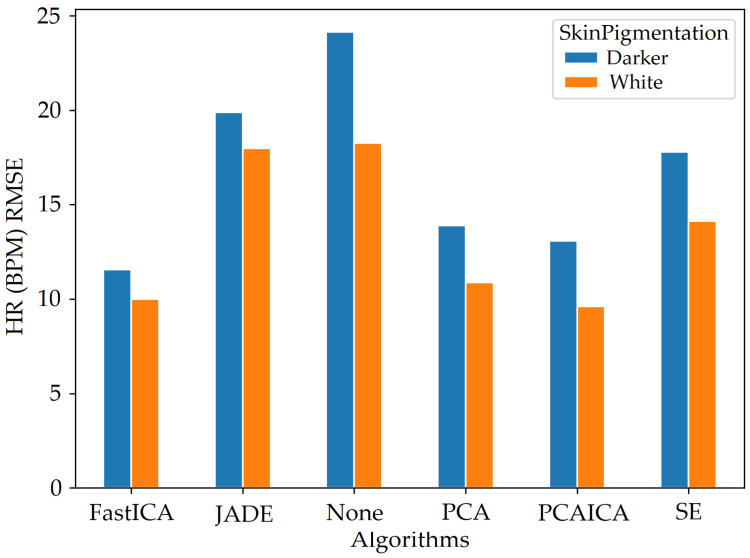
HR (BPM) RMSE values for participants with darker and white skin pigmentation. Plot shows RMSE HR (BPM) values averaged for resting and active states measured over participants with darker and white skin pigmentation.

**Figure 22:**
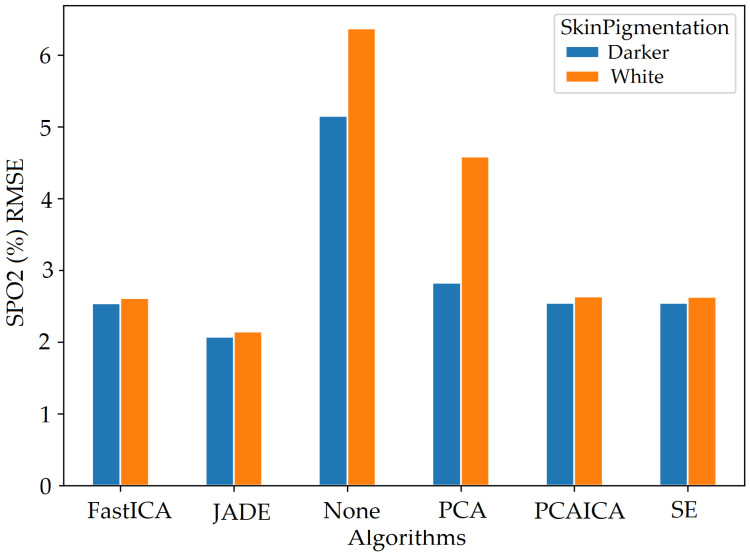
$SPO_{2}$ (%) RMSE values for participants with darker and white skin pigmentation. Plot shows RMSE HR (BPM) values averaged for resting and active states measured over participants with darker and white skin pigmentation.

The system was optimised to reduce skin pigmentation bias using data obtained from participants with different skin colours. Various noise reduction algorithms such as FastICA, PCA, JADE, and SE, were applied on the data and their corresponding RMSE values were compared as shown in Figure 19 for HR and Figure 20 for $SPO_{2}$. Figure 19 shows evaluation measures for white (right side of the image) and darker (left side of the image) skin pigmentation participants. Figure 19 shows that for participants with white skin pigmentation, FastICA, PCA, and PCAICA produced low RMSE values of 6.5, 7.53, and 6.7 compared to the rest of the algorithms, these values are also presented in Table 6. Similarly FastICA and PCAICA produced low RMSE values of 9.1 and 12.32 for participants with darker skin pigmentations as shown in Figure 19 and presented in Table 6. Averaged RMSE values for resting and active states for darker and white skin participants is shown in Figure 21 for HR and Figure 22 for $SPO_{2}$. No differences were found due to age or gender; however, parameters such as having a beard, which is common among men, and makeup among any gender can impact the results, which are discussed in Section 9 and Section 8.

**Figure 23:**
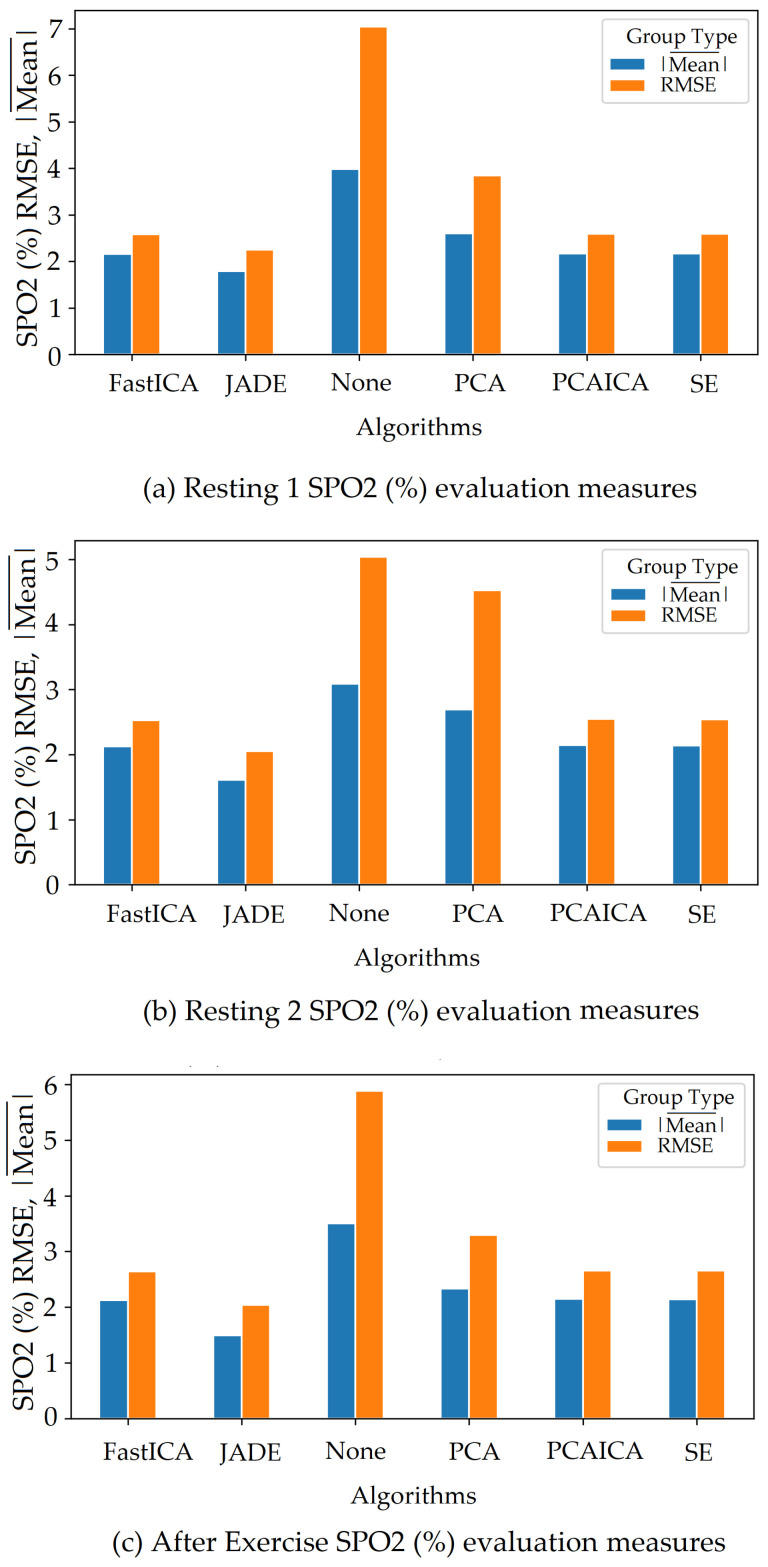
$SPO_{2}$ (%) RMSE values for all participants for resting and active states. The plots show MeanAbs (mean absolute error $|\bar{mean}|$) and RMSE for different noise reduction algorithms where None has the highest RMSE compared to rest of the algorithms. The signal data were also processed with no noise reduction algorithm, which is represented by ‘None’ on the plot and compared with rest of the noise reduction algorithms.

**Figure 24:**
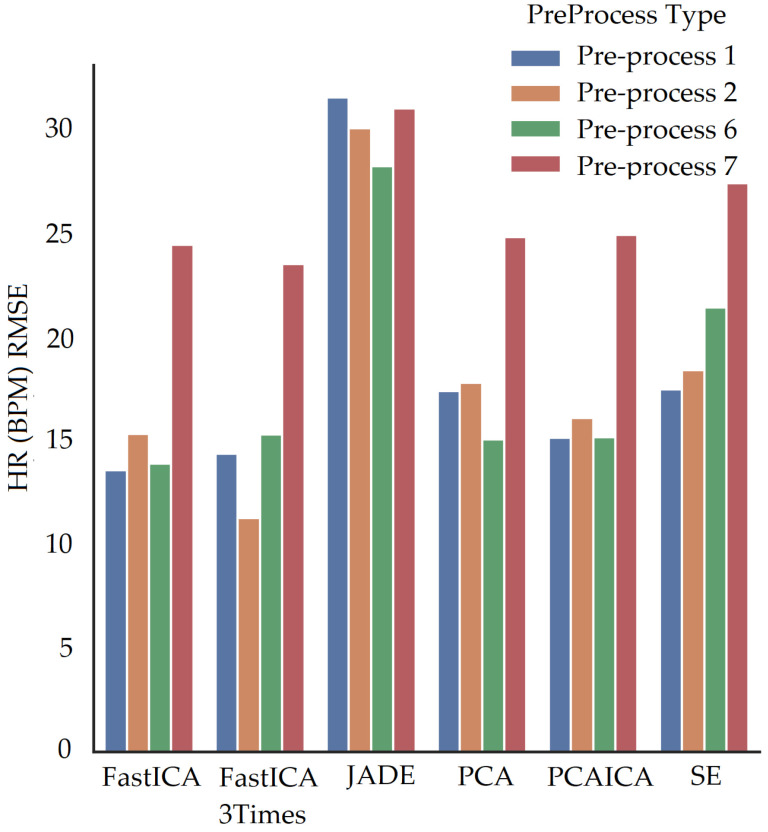
Pre-processing techniques applied on darker skin participants data in combination with algorithms. *Y*-axis shows HR (BPM) RMSE for darker skin participants and *X*-axis shows noise reduction algorithms applied with different pre-processes.

**Figure 25:**
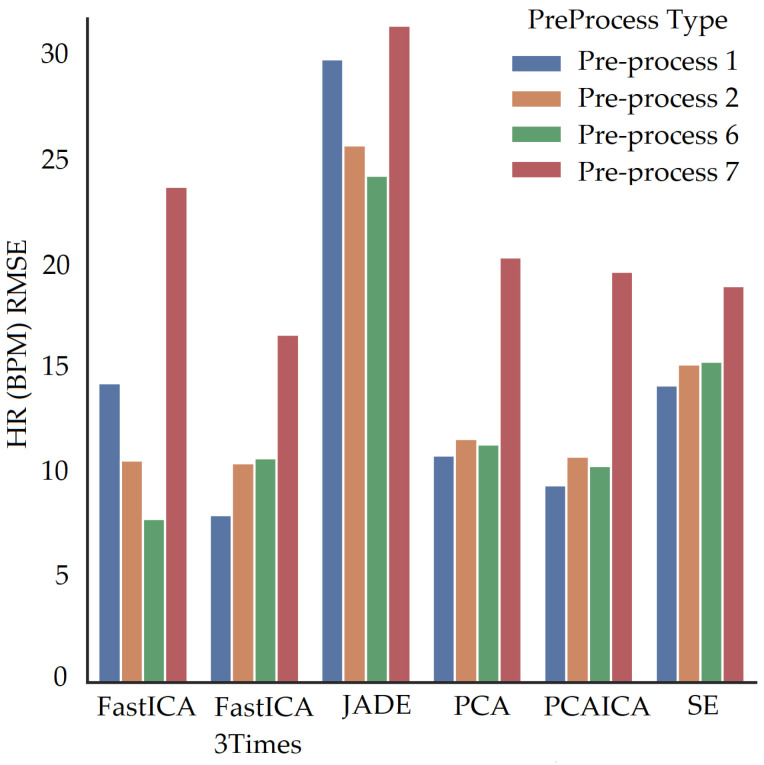
Pre-processing techniques applied on white participants data in combination with algorithms. *Y*-axis shows HR (BPM) RMSE for white skin participants and *X*-axis shows noise reduction algorithms applied with different pre-processes.

FastICA over all participants produced the lowest RMSE of 7.8 and a higher r correlation r-value of 0.85 compared to other algorithms (as shown in Table 6 and in Figure 19). For $SPO_{2}$ (%), all algorithms produced similar RMSE of approximately 2.5 as shown in Figure 23. Jade produced the lowest RMSE of 2.0 compared to the rest of the algorithms for resting and active states; however, Jade did not perform as well for obtaining HR as shown in Figure 18, which shows Jade and applying no algorithm on the data has the highest RMSE values. FastICA and PCA were also applied multiple times on the data for each channel ($S=s_{channel}$ where channel means R, G, B, Gy, and IR) at a time or all five components at the same time ($S=s_{R},s_{G},s_{B},,s_{Gy},s_{IR}$); however, applying PCA on the data more than once did not impact signal strength and FastICA applied more than three times also did not improve the signal data. RMSE comparison of FastICA applied once and three times for participants with darker skin pigmentation is shown in Figure 24 and for white skin is shown in Figure 25.

For participants’ with darker skin pigmentation, FastICA with a combination of pre-processing technique 6 or FastICA applied three time with pre-processing technique 2 produced the lowest RMSE compared to the rest of the algorithms as shown in Figure 24. Compared to that for white skin pigmentation participants, FastICA produced low RMSE for pre-processing technique 6 as shown in Figure 24. Over all participants pre-process 6 produces the lowest RMSE. Pre-processing technique 7 for participants produced low RMSE only when the fps of data were less than 15, which is detailed in Section 10.

## 6. Signal-to-Noise Ratio (SNR) by Regions of Interest (ROI) size and Channel Type

**Figure 26:**
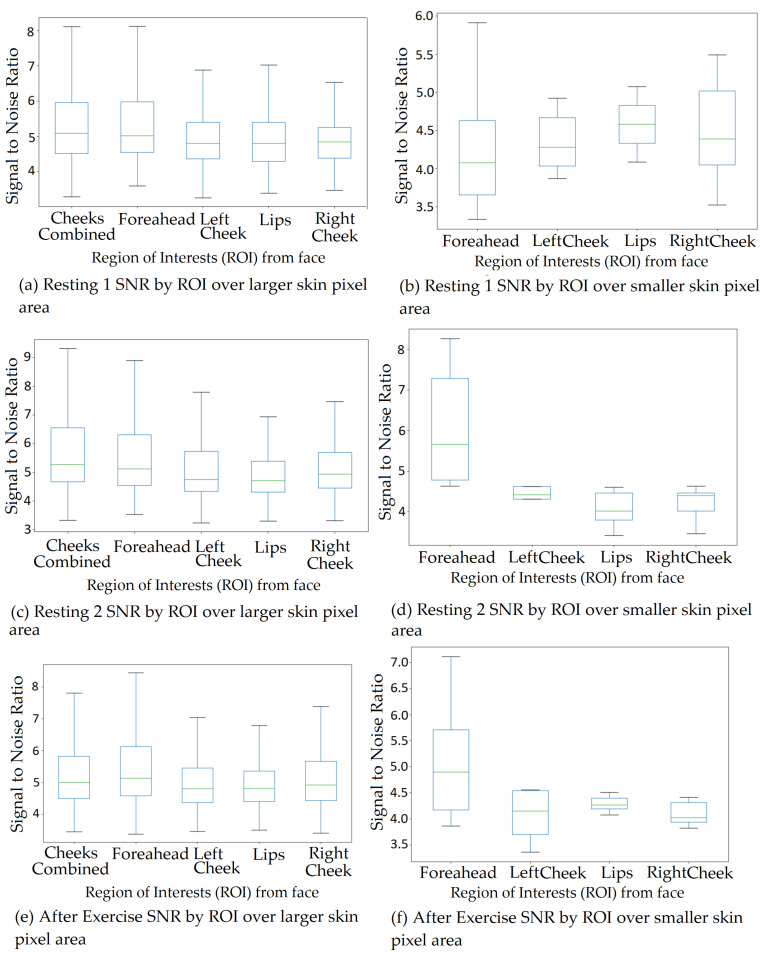
SNR by ROI over larger skin pixel area. SNR for ROIs over all participants showing comparison between larger and smaller skin pixel areas. The comparison shows that larger area of skin pixels generate a higher SNR compared to smaller pixel areas.

**Figure 27:**
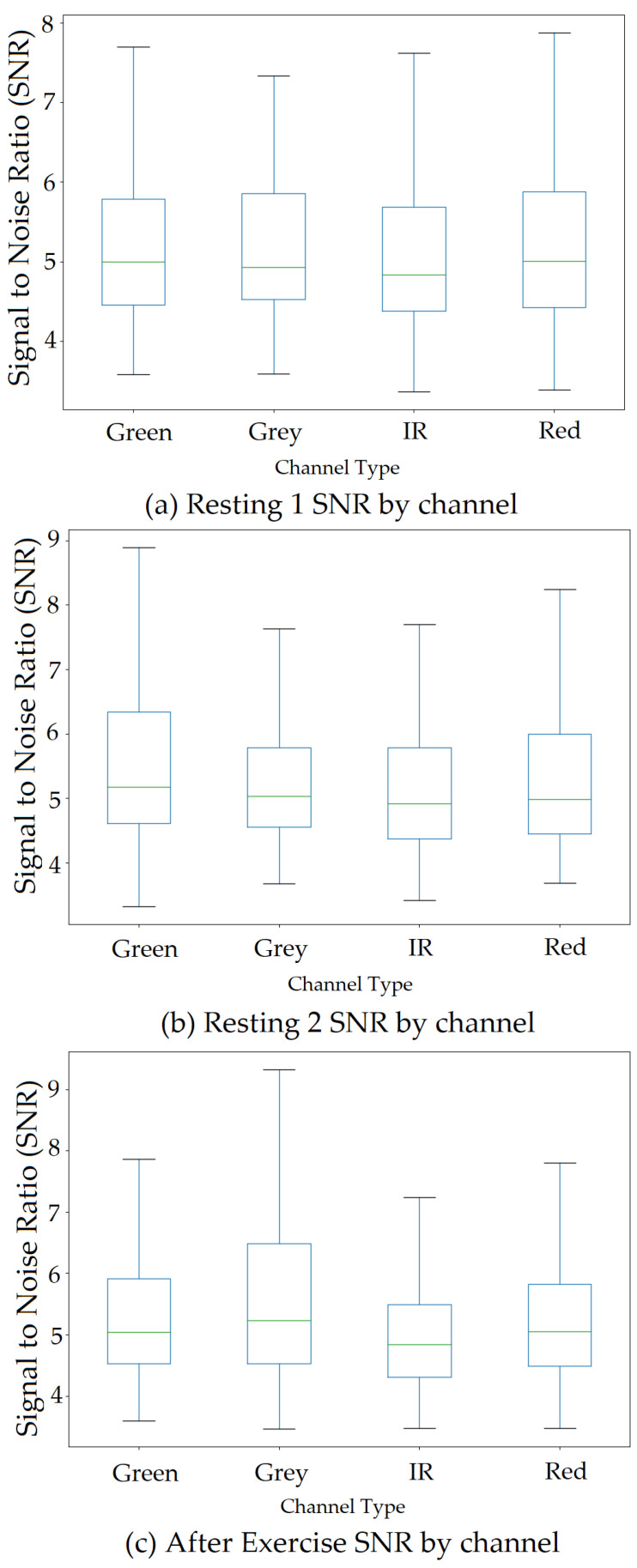
SNR by Channel Type. SNR by channel for all participants.

Initially, four ROIs (excluding combined cheeks with nose) were used; However, since participants were moving in front of the camera, another region from the centre of the face would be useful to add and to increase the likelihood of obtaining the best SNR. SNR was calculated for ROI with a larger and smaller (pixel) size. ROI with larger skin pixels provides better SNR, which helps extract a better signal from the collected dataset compared to that of the smaller skin pixel area. The box plot showing SNR comparison between larger and smaller skin pixel ROI shown in Figure 26 where the left side represents larger skin pixel SNR and right side represent plots showing SNR for smaller skin pixel area. The SNR was calculated by dividing frequency peak value of each channel by averaged signal that has been passed through FFT. This was obtained for each ROI and all channels of that ROI. SNR was also calculated by channel types shown in Figure 27. Researchers previously have used a single channel and ROI to calculate HR; however, only one channel or ROI cannot provide the best SNR values, and in a real-life home environment, it is necessary to monitor and process all ROI and channel signal data to select vital values with the highest SNR. Since ICA also extracts components using a random mixing matrix random, it is important to observe all channel signal outputs obtained after applying any algorithm, as opposed to previous research. This will ensure accurate readings depending on the SNR. Using all channels and ROIs to select the most optimal signal, which provides the best SNR produces the most accurate results.

## 7. Window Comparison

**Figure 28:**
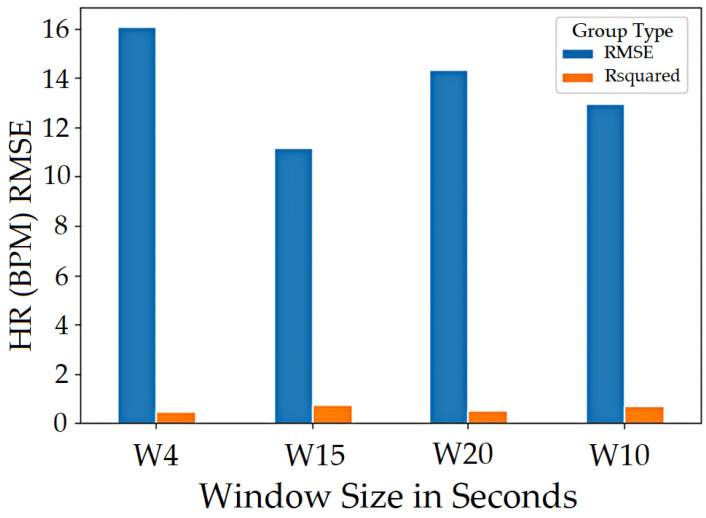
Window RMSE comparison. HR (BPM) RMSE comparison between different window sizes for all participants.

**Figure 29:**
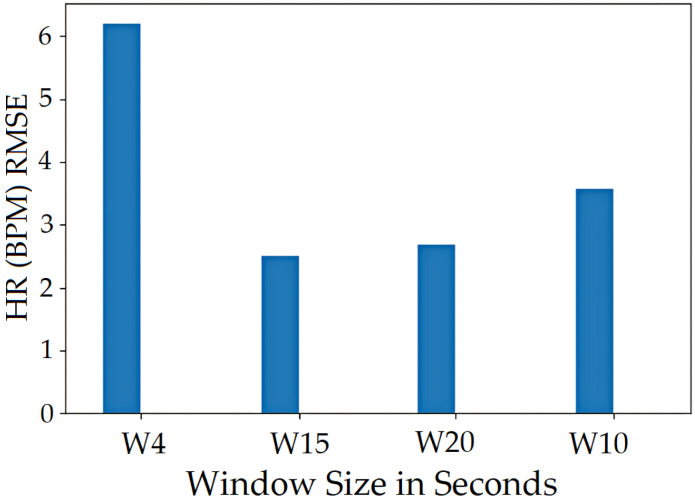
Window RMSE comparison. $SPO_{2}$ (%) RMSE comparison between different window sizes for all participants.

**Figure 30:**
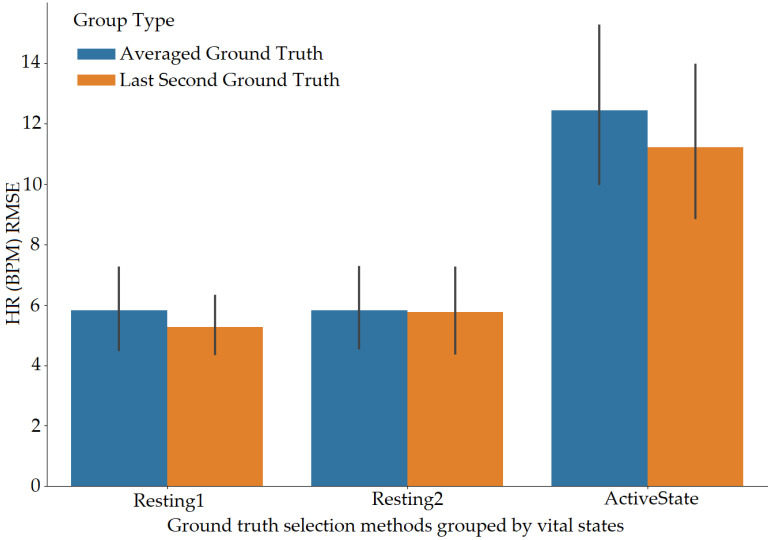
Ground truth selection method. HR (BPM) RMSE comparison between ground truth obtained using averaging method (average value obtained for a specific window size, for example, 4 s window,) and selecting last-second method (latest value from the window size) grouped by resting and active states.

The vital values were measured for participants over the entire signal data and over window sizes. The entire signal data of 60 s are not the most accurate way to test the ARPOS system against ground truth. This was as many participants’ heart rates varied for each second, especially in the case of exercise, meaning comparing each second of the ground truth to the ARPOS obtained data would be required to validate the system’s accuracy, instead of comparing to an averaged vital reading of 60 s. Along with that, in real life scenario, the real time system would acquire the frames in the form of second windows and produce results accordingly. Data were calculated for different window sizes such as 4, 10, 15, and 20 s. To compare data from the ARPOS system to the ground truth, it is essential for the ground truth data to be in a similar format (window size). One method to obtain ground truth in windows size format was to average the vital signs for that specific window size (number of seconds). Another method where the last second vital signs from that window size was selected (latest value). The last-second values from the ground truth compared against the ARPOS obtained vital values produced slightly better (lower RMSE) than the average ground truth of the window size. The 15-s window size produces the lowest RMSE value (most optimal) results, showing that data from ARPOS system are closer to the ground truth as shown in Figure 28 (HR) and Figure 29 (SPO). Figure 30 shows RMSE comparison between ground truth obtained using averaging method and selecting last-second method grouped by resting and active states. The ARPOS system vitals were compared against the last second vital from the ground truth device.

## 8. MakeUp

**Figure 31:**
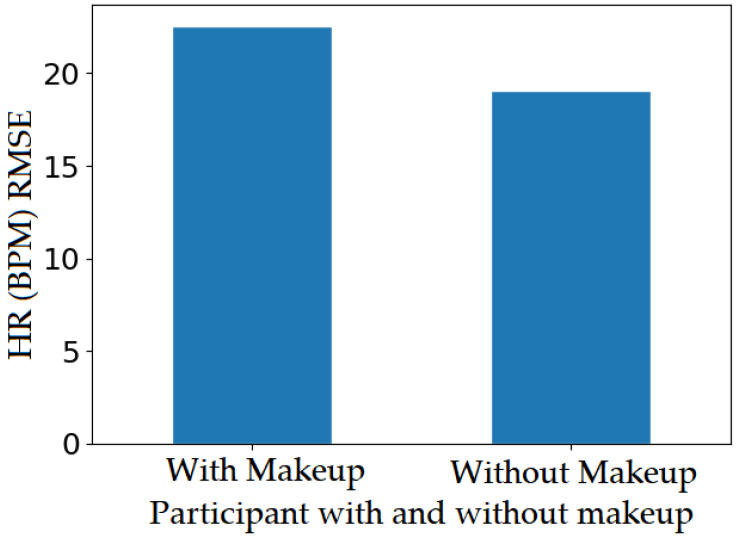
Makeup RMSE for HR (BPM) comparison. Participant with lipstick for lip region, where left bar shows RMSE with make up (PIS-3252) and without make up on right (PIS-3252P2).

The impact of makeup on the accuracy of an rPPG system has not been studies. In the ARPOS research study, one participant was wearing lipstick, which impacted the vital values obtained from lip region. It cannot be concluded since only one participant was wearing lipstick during the data acquisition; however, once the participant retook the study (same person) without lipstick, the RMSE for lips decreased, as shown in Figure 31. So far, no previous research has been identified that addresses the makeup impact on results obtained from rPPG system. This could be due to low participation from females (gender bias) or simply because it was conducted in a lab environment with a restriction such as no makeup; however, these factors are important to be studied as, in a real-life scenario, these factors may affect the accuracy of the system. In this research study, the ARPOS system chose another ROI (instead of lip) with comparatively better SNR than lips to obtain an accurate vital reading; therefore, the ARPOS system chooses the most optimal ROI to extract signal data, which has been tested to work in a real-life environment. The RMSE comparison shown in plot Figure 31 was only generated to show the impact of makeup. The participant was wearing nude colour lipstick; however, participants wearing darker lipstick such as red or black could produce a very high RMSE value. This factor would require further research.

## 9. Beard

**Figure 32:**
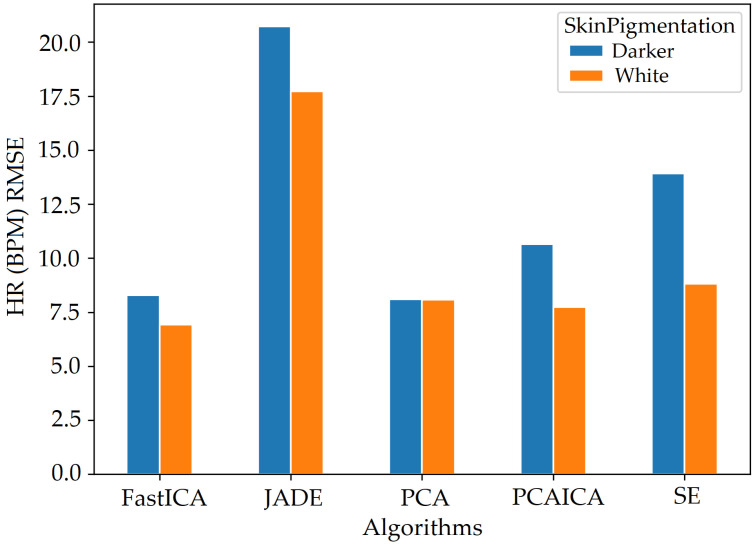
HR (BPM) RMSE for white and darker skin participants with beard. Figure showing RMSE bar plots where different noise reduction algorithms have been applied on data of participants (12.5%) with heavy beard of white skin and darker skin pigmentation.

**Figure 33:**
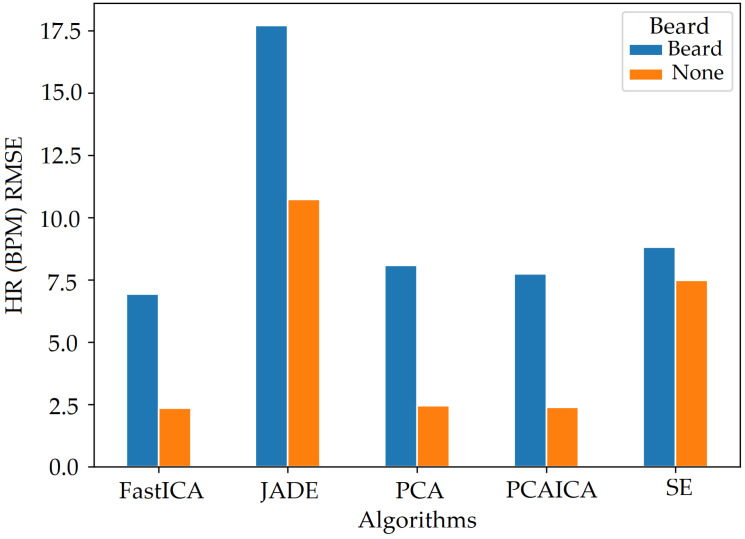
HR (BPM) RMSE for white skin participants with and without beard. Figure showing RMSE bar plots where different noise reduction algorithms have been applied on data of participants (5%) with heavy beard of white skin pigmentation.

**Figure 34:**
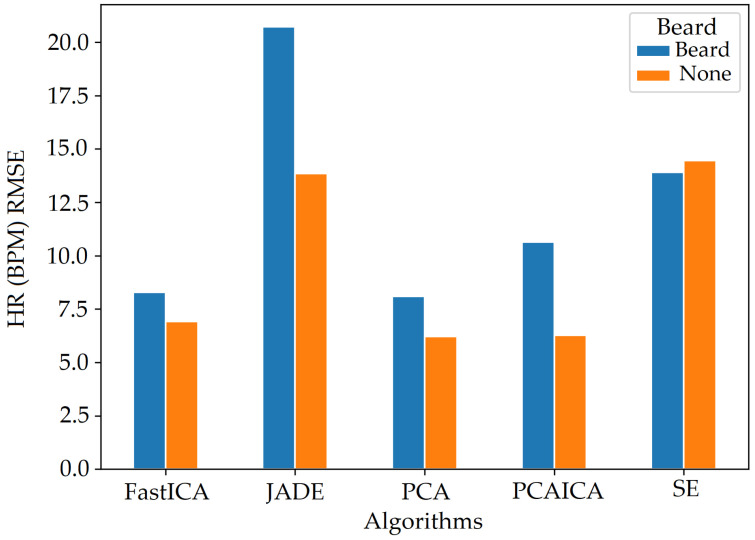
HR (BPM) RMSE for white skin participants with and without beard. Figure showing RMSE bar plots where different noise reduction algorithms have been applied on data of participants (7.5%) with heavy beard of white skin pigmentation.

Participants with a beard had higher noise compared to those who did not, which impacted the overall results. Figure 32 shows that darker skin pigmentation participants with a beard have higher HR (BPM) RMSE values compared to white participants. The RMSE values for participants with beard is also comparatively higher then those without beard for participants of white and darker skin pigmentation as shown in Figure 33 and Figure 34.

## 10. Impact of Variable FPS

Different hardware (low RAM and GPU processing power) and illumination (leading to longer exposure times) within participant’s home environments worldwide impacted the frames per second (fps). For example, participants with darker skin pigmentation in very dim light or participants with white skin in very bright light also impacted fps and therefore the sampling frequency of the heart rate (HR) signal corresponding frequency resolution, which therefore affects the error of the HR measurement. It is therefore important to cater for a variable fps; however, in previous systems a constant fps value of either 30 or 15 is selected as the equipment is used within a controlled environment or lab setting. Since participants deployed the study setup on their devices, the RAM utilised varied for each system; therefore, the system design was updated to handle different types of data, including updating fps for each window size depending on the participant’s colour and IR data.

This is important as constant values for all participants with different fps, frequency, and number of samples would require parameters to be dynamic for each second window in order to calculate vital signs. For example, if a participant has an fps of 30 for colour and IR and another participant with 15 colour fps and a variable fps ranging from 10 to 24 for IR would be require appropriate interpolation and re-estimation for each window size for variable fps to process differently as the signal obtained from both data will not have the same number of samples. Hence, the program needs to be able to process all the fps groups as per the input and apply techniques accordingly.

**Figure 35:**
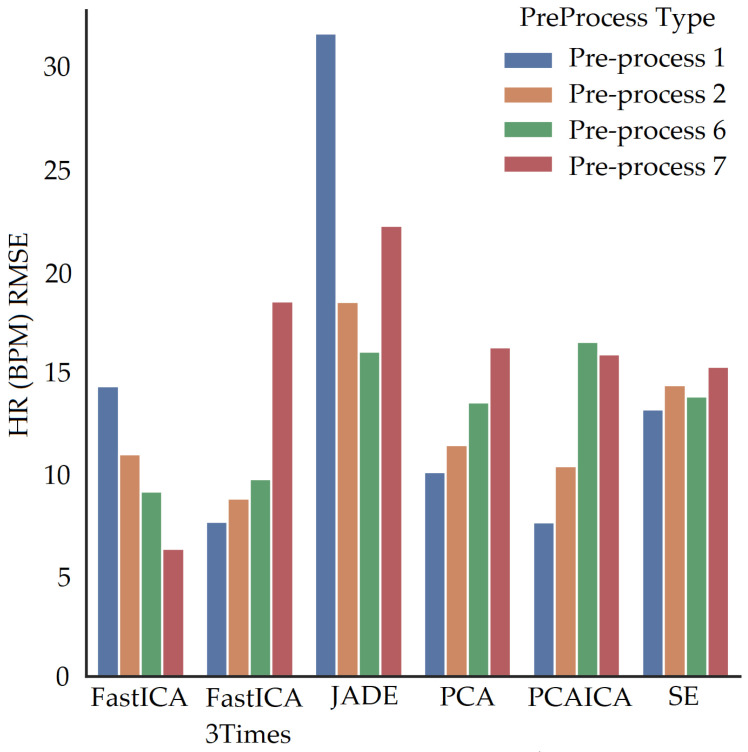
HR (BPM) RMSE comparison for pre-processing techniques with noise reduction algorithms applied for participants with fps lower than or equal to 15.

**Figure 36:**
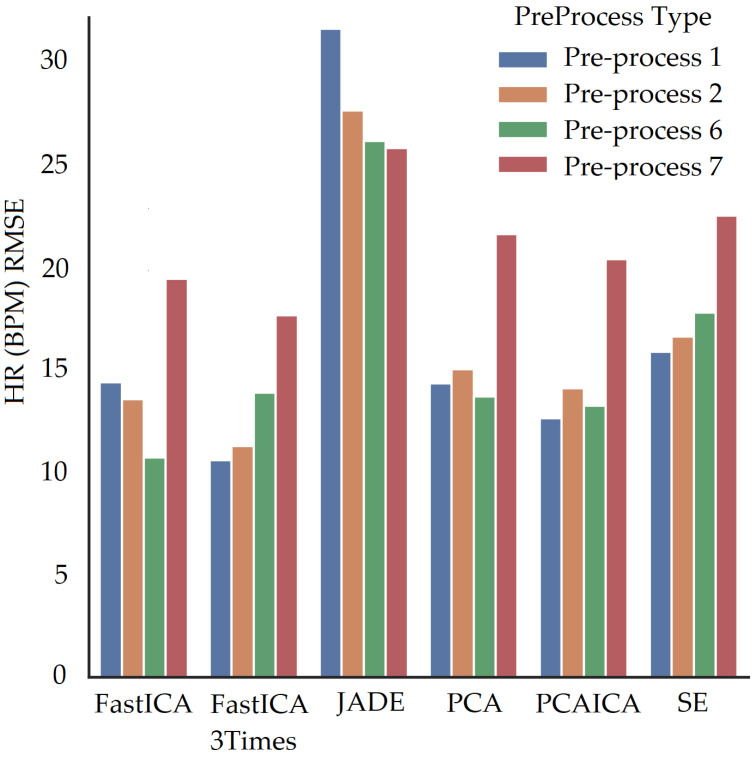
HR (BPM) RMSE comparison for pre-processing techniques with noise reduction algorithms applied for participants with fps greater than 15.

When fps is lower than 15 for any channel data, pre-processing technique 7, in addition to noise reduction algorithms, produces vital signs closer to the ground truth (low RMSE value) as shown in Figure 35 where FastICA with pre-processing technique 7 produces lowest RMSE. The pre-processing technique 7 involves upsampling of data along with interpolation and detrending of data. RMSE comparison for pre-processing techniques with noise reduction algorithms for participants with fps greater than 15 is shown in Figure 36. Pre-process 2 and 6 over all participants produces lowest RMSE with FastICA and PCAICA with the lowest RMSE.

## 11. Retaken Studies

**Figure 37:**
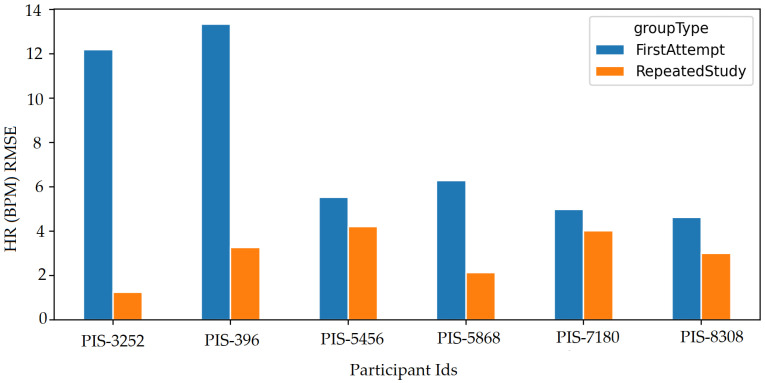
Participants retook the study for data with low fps. The retaken studies HR (BPM) RMSE has reduced quite a lot after fps has increased.

Some of the participants were asked to retake the study due to very low fps caused by very low illumination or low RAM; however, participant’s data with low fps or illumination have also been evaluated in addition to the retaken study. Figure 37 shows participant’s data before retaking the study and the improved results after they retook the study with improved illumination (not in dim lights) and fps (increased RAM by closing multiple applications, not in use on the PC).

## 12. Computational Expense

**Figure 38:**
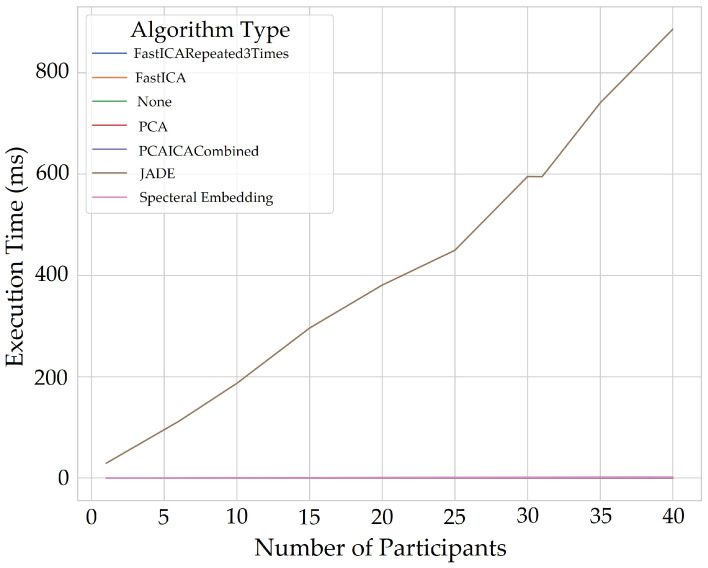
Time taken to process participants data grouped by algorithms (for five components at a time). The plot shows different noise reduction algorithms execution time, including the entire process of obtaining the vital signs such as spatial pooling, pre-processing, noise reduction algorithm, FFT, and filtering. Jade seems to take the most execution time compared to rest of the algorithms. Rest of the algorithms have execution time of less than 5 ms, which makes it look like its only one line. Zoomed in view of the plot where rest of the noise reduction algorithms execution time is shown for individual components in Figure 39 and for all components in Figure 40.

Measuring computational expensive is essential to identify the execution time of different algorithms that can help select the most optimum algorithm to implement for a real-time application. This also helps us identify the hardware required for monitoring as many people as possible and as regular as possible in real time. There is also a lack of discussion regarding computation expensive in previous literature; therefore, computational expense for multiple participants for extracting vital signs was also calculated. The system used the UWP app to acquire 64 s of data for each participant, detected the face area for colour, mapped to IR and depth images and sent ROI to the server, which used a memory of 2 GB. The memory occupied by an individual participant for the entire process of extracting vital signs (excluding ROI extraction) for 60 s is 555 MiB; however, in real-time only 15-s of data would be processed at a time. The queue would follow FIFO (first in first out) method meaning for each new data frame arriving, the initial ROI data would be released from the memory, hence maintaining the 15 s window and re-calculating the vital signs. The system took the longest time to process data with Jade, FFT, filter, and extracting the vitals compared to the rest of the algorithms shown in Figure 38 which takes up to 600 ms (milliseconds) for 30 participants.

**Figure 39:**
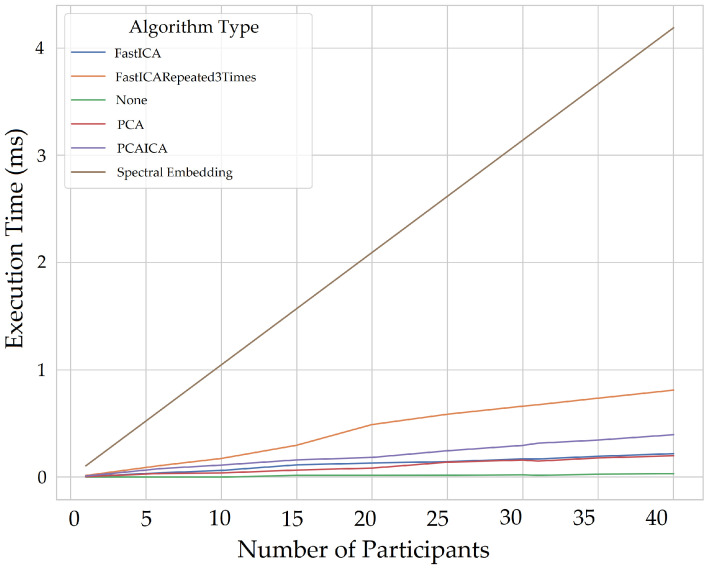
Time taken to process participants data grouped by algorithms (for single component at a time). The plot shows different noise reduction algorithms execution time, including the entire process of obtaining the vital signs such as spatial pooling, pre-processing, noise reduction algorithm, FFT, and filtering. Processing individual components individually takes double the time compared to processing all the components at the same time as shown in Figure 40.

**Figure 40:**
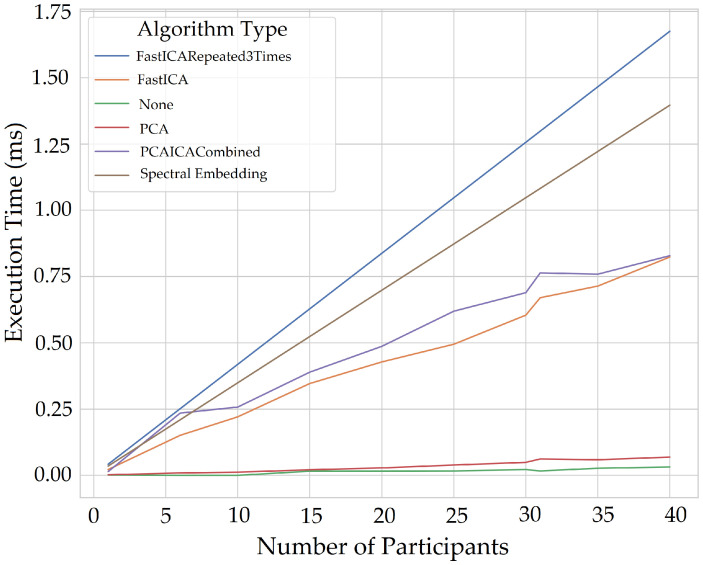
Time taken to process participants data grouped by algorithms (without Jade and for five components at a time). The plot shows different noise reduction algorithms execution time, including the entire process of obtaining the vital signs such as spatial pooling, pre-processing, noise reduction algorithm, FFT, and filtering. Processing all the components at the same time takes 50% reduced time as compared to processing each component individually as shown in Figure 39.

The execution time for algorithms also increases when the entire signal is processed for all five components compared to processing each component at a time. Figure 39 and Figure 40 shows plots without Jade to demonstrate time execution by different algorithms when each component is processed (one component at a time) compared to processing all components at a time. Processing each component at the same time using algorithms takes double the time (4 ms) compared to that of processing all components at a time (1.75 ms) for all participants. The plots below show number of participants on *X*-axis and execution time in milliseconds. The *X*-axis starts for one participant and the step increments by five to evaluate execution time taken by each noise reduction algorithm.

## 13. Key Research Contributions

A health monitoring system (ARPOS) was designed and developed by the researcher to take vitals including HR and $SPO_{2}$ in a non-invasive and unobtrusive manner. The system was tested and improved compared to the previous system for participants of different skin colours, and gender-related factors (such as makeup and beard) to see which techniques and methods produce the most optimal results in comparison to ground truth. In addition to those computational expensive were also investigated, which is missing in the previous system. This is the first system that has been validated and optimised within the participant’s home environment and was also deployed and operated by them.

The anonymous data acquired during the ARPOS research study has been made open-source meaning shared and made available to all researchers using open data formats. This enables other researchers to build on the results and accelerate the development of new research studies. In addition to that, the research papers are also published in open-source format. This is the first open-source dataset made available that was collected from participants’ homes, i.e., real-life environments worldwide. The data set is available at https://doi.org/10.5281/zenodo.6522389 (accessed on 27 June 2022) [72].

The infrastructure of the ARPOS system was designed by the researcher in a way that future developers and researchers can build upon the system to make it deployable in homes, care homes, and hospitals, as it is scalable (technically and otherwise) and can be conducted remotely or face to face. The project code is available on GitHub, so researchers can replicate and build on this work. The ARPOS GitHub project is available at https://github.com/PirehP/ARPOSpublic (accessed on 27 June 2022). The ARPOS system has been developed and tested for potential skin bias, age groups, gender, different light within home environments, makeup, beard, and other parameters that impact results obtained from data in real-life environments. Other parameters include different hardware capabilities (RAM, CPU, GPU), fps, and computational expense for multiple participants are discussed in the results section.

The successful ARPOS system is proof that participants were easily able to use and install the system within their homes. New knowledge was found from each research study contributing toward the advancement of (health-related) smart homes. The researcher designed all the studies to be user-centric, that is, considering user viewpoints. This methodology, design, and development will help developers and researchers to create systems that cater for the needs of people around the world. No similar methodology has been implemented so far, where an rPPG system has been deployed and validated by participants within their homes worldwide from different cultural and socioeconomic backgrounds. The methodology and experiment protocol is published and available at https://dx.doi.org/10.17504/protocols.io.n2bvj6zkxlk5/v1 (accessed on 27 June 2022) and is detailed in the methodology section of this paper.

## 14. Recommendations and Limitations

The next step for the research would be to stress test and validate the ARPOS system in a clinical setting such as a respiratory ward so as to test a variety of HR and $SPO_{2}$ among patients. It would be interesting to see how the different techniques and methods perform in a clinical setting compared to home environments of people where participants are mostly healthy. In addition to that, the system could be designed to use another affordable camera or develop a custom camera with sensors to take colour, IR, and depth data. This will make it accessible to rural and urban areas around the world (lower or middle-income countries and/or areas with limited access to the internet) and cost-effective, enabling this to be a system affordable by all. The system would be accessible and based on cross-platform open-source technologies and not restricted to a single camera system. Physiological human health monitoring requires not only HR and $SPO_{2}$ but also body temperature, blood pressure, and glucose monitoring. These different vitals could be added to the system to encompass other factors required to monitor health and well-being.

Automatically recalling historical data of individuals using facial recognition could be integrated into the ARPOS System. This will enable the system to create a history of a patient’s vitals and can be used to alert, identify trends, and enable detailed health status monitoring. For example, ARPOS records the history of a person’s vitals before they go to the toilet and appends (link their recently acquired vitals) to the same person’s data when they return and maintain vital history. The system can also be improved by increasing the limit of monitoring 6 participants to 10 or more. Automatic skin pigmentation selection can be implemented in the ARPOS system, so as to apply pre-processing techniques according to participants skin tone to reduce noise from the signal. Further studies are required to further minimise bias by testing different skin pigmentations such as black participants and a larger dataset for different participants from various parts of the world. Further, due to the recent pandemic, face masks have become a common part of people’s life. Even though the restrictions in certain parts of the world are relaxing, at the time of submission of this thesis, people continue to wear face masks, which could hinder the face detection and health monitoring of people. Improved face detection techniques could be employed to identify ROIs from the face with a facemask. Further studies also need to be conducted on faces with different lengths of beard using different noise reduction algorithms in addition to ROI extraction from the face so as to minimise areas, which do not provide a signal such as teeth and beard. Further, participants with different levels of makeup could also be involved in the research study to test the system, for example, a participant with lipstick, foundation, and concealer.

## 15. Conclusions

In conclusion, a robust study was conducted to measure vital signs (HR and $SPO_{2}$) in a non-contact-based manner from healthy participants worldwide. The ARPOS system was deployed and operated by participants proving its ease of use. The system was validated on different devices with varied RAM and processing power. This was the first study designed to validate an rPPG system’s accuracy within participants’ home environments (real-life scenario) and investigating various factors that impact the accuracy of reading vital signs from participants’ faces. These factors include various home environment backgrounds, illumination, skin pigmentation, makeup, and beards, computing hardware, and impact of fps on vital signs.

The ARPOS study also found that frames per second (fps) impact the quality of signal data. If fps is greater than 15 then pre-processing technique 6 (Detrend, interpolate, hamming, and normalising the signal) helps extract vital values closer to ground truth in combination with FastICA or PCAICA. Whereas if the fps is less than 15 then pre-processing technique 7 (Detrend, upsample, interpolate, hamming, and normalising the signal) produced vital values closer to ground truth in combination with FastICA and PCAICA. For all participants, pre-processing techniques 6 on the signal with FastICA produced the lowest RMSE of 7.8 for HR (BMP) with the r-correlation value of 0.85 and standard deviation error of 0.024 and RMSE 2.3 for $SPO_{2}$ (%). If fps was lower than 15, pre-processing technique 7 was applied in addition to FastICA to produce similar results. Whereas for the active state, PCAICA produced the lowest RMSE of 13 for HR and 2.5 for $SPO_{2}$ for all participants.

This novel system could be deployed within care homes, hospitals, communities, and private homes where health concerns can be immediately recognised and acted upon. Real-time communication of these vital signs can flow between the subjects and the carers, emergency services, or private users while catering to the ethical and privacy needs, and without the requirement to store personal data. The system could be utilised for vulnerable people who are isolated at home, for example, those with dementia, or for those who may benefit from continuous monitoring in care home and hospital settings. In turn, this could help reduce pressure on emergency and non-emergency health care providers, reducing cost, and providing triage and urgent intervention to those who need it the most. A current healthcare priority remains around COVID-19. In this context, those with emerging respiratory distress would be recognised without the need for contact, with abnormal observations relayed through the ARPOS without the infection transmission risks. In a larger setting, either in primary care or within hospitals, patients awaiting triage for further care could be continuously screened to pick up early changes in vital signs, and thereby trigger earlier intervention as required. Finally, in the non-healthcare setting, the technology could potentially also be used to scan larger crowds or gatherings in real time to identify potentially dangerous rhythms of heart rate, breathing pattern or oxygen levels, or temperature, and so trigger rapid earlier intervention.

## Figures and Tables

**Table 1:** Previous systems acquiring only HR BPM (lab based).

Year	Author	Participants	SD ^1^	RMSE ^1^	R ^1^
2010	Poh et al. [19](Sitting still)	12	2.29	2.29	0.98
2010	Poh et al. [19](with slight movement)	12	4.59	4.36	0.95
2010	Poh et al. [19] reported by Hassan et al. [20]	12	12.82	21.08	0.34
2010	Poh et al. [19] reported by Waqar et al. [21]	12	14.57	17.70	0.33
2011	Poh et al. [22]	12	0.83	2.29	1.00
2011	Poh et al. [22] reported by Hassan et al. [20]	20	12.66	14.01	0.44
2011	Poh et al. [22] reported by Waqar et al. [21]	5	18.12	18.02	0.14
2013	Monkaresi et al. [23](ICA)	18	25.54	35.31	0.53
2013	Monkaresi et al. [23](ICA+KNN)	18	4.33	4.33	0.97
2013	Monkaresi et al. [23](ICA+KNN+Regression)	18	13.70	13.69	0.58
2014	Li et al. [24] VideoHR database	10	0.72	1.27	0.99
2014	Li et al. [24] MAHNOB-HCI database	27	−3.30	7.62	0.81
2014	Li et al. [24] reported by Hassan et al. [20]	20	9.53	12.47	0.53
2015	Kumar et al. [25] (still)	12	-	15.74	-
2015	Kumar et al. [25] (reading)	5	-	55.34	-
2015	Kumar et al. [25] (watching video)	5	-	97.51	-
2015	Kumar et al. [25] (talking)	5	-	67.08	-
2022	Zheng et al. [26] (Low illumination)	40	5.64	7.63	0.85
2022	Zheng et al. [26] (average illumination)	40	4.55	6.28	8.75
2022	Zheng et al. [26] (high illumination)	40	3.54	5.09	0.86
2022	Zheng et al. [26] (unbalanced illumination)	40	4.96	7.33	0.84
2022	Zheng et al. [26] (slight head movement)	40	5.95	7.03	0.85

^1^ Previous systems listed acquired data using lab-based studies (fixed environment) and did not acquire *SPO*_2_. *SD*→*Standard Deviation*, *RMSE*→*Root Mean Square Error, r*→*r-correlationvalue*.

**Table 2:** Previous systems acquiring only $SPO_{2}$.

Year	Author	Participants	RMSE ^2^
2021	Mathew et al. [27] (Model1 PD)	14	3.07
2021	Mathew et al. [27] (Model1 PU)	14	2.16
2022	Casalino et al. [28] (Still)	10	1.879
2022	Casalino et al. [28] (talking)	10	1.188
2022	Casalino et al. [28] (slight rotation)	10	1.881
2022	Casalino et al. [28] (some rotation)	10	1.063
2016	Van Gastel et al. [29] (Still)	4	1.33
2016	Van Gastel et al. [29] (some movement)	4	1.64

^2^ Previous systems listed acquired *SPO*_2_. *RMSE*→*Root Mean Square Error*.

**Table 3:** Participant Information.

Description	Total	Percentage
Participant’s Country	40	100%
United Kingdom	23	57.5%
Pakistan	16	40%
Malta	1	2.5%
Participant’s Gender	40	100%
Female	25	62.5%
Male	15	37.5%
Participant’s Age	40	100%
18–30	24	60%
30–40	8	20%
40–50	5	12.5%
51–60	1	2.5%
61 or above	2	5%
Participant’s Skin Pigmentation	40	100%
White	21	52.5%
Asian White	1	2.5%
Brown	14	35%
Darker	4	10%
Black	0	0%
Participant’s Ethnicity	40	100%
European	21	52.5%
Asian (South)	18	45%
Asian (Other)	1	2.5%
Participants asked to repeat research study from Europe	3	7.5%
Participants asked to repeat research study from Asia	3	7.5%
Participants consented but did not participate	3	7.5%
Participants data acquisition stopped due to health concern	1	2.5%
Participants consented to the research study (including repeat participants)	40	100%
Total participant’s data analysed (including repeated studies)	40	100%

Participant information categorised by country of residence, gender, age group, skin pigmentation, ethnicity, and participation in the research study.

**Table 4:** Pre-processing Techniques Combinations.

Pre-Processing Type	Description
Type 1	No processing
Type 2	only normalise signal
Type 3	Interpolate, apply hamming, smooth, median filter, and normalise
Type 4	Detrend, interpolate, apply hamming, smooth, median filter, and normalise
Type 5	Interpolate, apply hamming, and normalise
Type 6	Detrend, interpolate, apply hamming, and normalise
Type 7	Detrend, upsample, interpolate, apply hamming, and normalise

**Table 5:** ARPOS system $\bar{mean}$ and $|\bar{mean}|$ for vitals over all participants.

Vital Type	State Type	$\bar{\mathrm{mean}}$	$|\bar{\mathrm{mean}}|$
HR (BPM)	Resting state	±0.5	±5.5
HR (BPM)	Active state	±1.88	±9.3
$SPO_{2}$ (%)	Resting state	±2	±2
$SPO_{2}$ (%)	Active state	±2	±2

**Table 6:** HR evaluation measures from the ARPOS system.

Analysis Type	SD(SE) ^4^	RMSE ^4^	r ^4^
All participants, resting with FastICA	0.018	**7.8**	**0.85**
All participants, active with PCAICA	0.014	15	0.75
White participants, resting with FastICA	0.015	6.5	0.87
White participants, resting with PCAICA	0.015	6.7	0.86
White participants, resting with PCA	0.017	7.53	0.81
Darker participants, resting with FastICA	0.0289	9.1	0.78
Darker participants, resting with PCAICA	0.027	12.32	0.64
Darker participants, resting with PCA	0.032	12.97	0.73
White participants, active with FastICA	0.018	18.5	0.72
Darker participants, active with FastICA	0.028	17.9	0.73
White participants, active with PCAICA	0.017	15.45	0.77
Darker participants, active with PCAICA	0.025	14.59	0.71

^4^ Resting states shown in this table are averaged values for resting state 1 and resting state 2 for participants. *SD(SE)*→*Standard Deviation Error, RMSE*→*Root Mean Square Error, r*→*r-correlation value*.

**Table 7:** $SPO_{2}$ evaluation measures from the ARPOS system.

Analysis Type	RMSE ^5^
All participants (resting state) using FastICA	**2.5**
All participants (active state) using PCAICA	**2.5**
White participants from Europe (resting state) using FastICA	2.5
Darker participants from Asia (resting state) using PCAICA	2.27
White participants from Europe (active state) using PCAICA	2.3
Darker participants from Asia (active state) using PCAICA	2.7

^5^ Resting states shown in this table are averaged values for resting state 1 and resting state 2 for participants. *RMSE*→*Root Mean Square Error*.

## Data Availability

The corresponding anonymous extracted regions of interest (forehead, cheeks, and lips) are available at https://doi.org/10.5281/zenodo.6522389 (accessed on 27 June 2022). The code used to post process the data is available at https://github.com/PirehP/ARPOSpublic (accessed on 27 June 2022). Experiment protocol followed for this research study is available at https://dx.doi.org/10.17504/protocols.io.n2bvj6zkxlk5/v1 (accessed on 27 June 2022).

## Abbreviations

The following abbreviations are used in this manuscript:

HR	Heart Rate
$SPO_{2}$	blood oxygenation level
RMSE	root mean square error
SD/SE	standard deviation/standard deviation error
FPS	frames per second
ARPOS	automated remote pulse oximetry system
rPPG	remote photoplethysmography
BPM	beats per minutes
PPG	photoplethysmography
ECG	electrocardiogram
Hb	oxy-haemoglobin
deoxy-Hb	deoxy-haemoglobin
KNN	K-nearest neighbour
NLMS	normalised least mean square
SNR	signal-to-noise ratio
LE	Laplacian eigenmap
AR	autoregressive
CCD	charge-coupled device
MM	millimetres
SE	spectral embedding
NM	nano meter
ICA	independent component analysis
UWP	Universal Windows Platform
R	red
G	green
B	blue
IR	infrared
Gy	grey
PCA	principal component analysis
JADE	joint approximation diagonalisation of eigen-matrices
FFT	fast Fourier transform
